# Lateral opening site of human oligopeptide transporter 2 plays a key role in the interaction with polymyxins

**DOI:** 10.7554/eLife.111387

**Published:** 2026-10-29

**Authors:** Xukai Jiang, Yining Luo, Mohammad AK Azad, Limei Xu, Min Xiao, Tony Velkov, Kade D Roberts, Visanu Thamlikitkul, Qi Tony Zhou, Fanfan Zhou, Jian Li

**Affiliations:** 1 National Glycoengineering Research Center, Shandong University Qingdao China; 2 Molecular Drug Development Group, Sydney Pharmacy School, Faculty of Medicine and Health, The University of Sydney Sydney Australia; 3 Biomedicine Discovery Institute, Infection Program, Monash University Melbourne Australia; 4 Division of Infectious Diseases and Tropical Medicine, Department of Medicine, Faculty of Medicine Siriraj Hospital, Mahidol University Bangkok Thailand; 5 Department of Industrial and Molecular Pharmaceutics, College of Pharmacy, Purdue University West Lafayette United States; 6 Save Sight Institute, Faculty of Medicine and Health, The University of Sydney Sydney Australia; Murang'a University of Technology Kenya; Iowa State University United States

**Keywords:** antibiotic resistance, drug discovery, hPepT2, polymyxin-induced nephrotoxicity, structure-interaction relationship, *E. coli*, Mouse, Other

## Abstract

Multidrug-resistant (MDR) Gram-negative bacteria pose a critical global health threat, while polymyxins remain a last-line therapy. However, their clinical use is limited by nephrotoxicity. Human oligopeptide transporter 2 (hPepT2) is a membrane transporter mediating the reabsorption of polymyxins in renal cells and contributes to their nephrotoxicity, but the molecular basis of their interaction remains unclear. Here, we investigated the structure-interaction relationship (SIR) of polymyxins with hPepT2 by integrating computational, chemical, and cell biology approaches. Bioinformatic modeling predicted an outward-facing hPepT2 structure and a potential transport pathway, with polymyxins interacting at the lateral opening, particularly E214, D215, D317, D342, and E622. Transporter mutagenesis and molecular analyses confirmed that D215 is critical for polymyxin binding, while other residues influence transporter turnover and/or expression. We subsequently synthesized polymyxin analogs with modifications at Dab1, Dab3, Dab5, and Dab9 of polymyxins, which reduced interactions with hPepT2. Notably, alanine substitution at Dab3 reduced nephrotoxicity in mice while retaining antibacterial activity. Overall, this proof-of-concept study demonstrates that the hPepT2-polymyxin SIR model provides a viable strategy for developing novel, safer lipopeptide antibiotics.

## Introduction

The World Health Organization has highlighted antimicrobial resistance as an urgent threat to global public health (Årdal et al., 2020; GBD 2021 Antimicrobial Resistance Collaborators, 2024; Morehead and Scarbrough, 2018). In particular, the Gram-negative ‘superbugs’ *Acinetobacter baumannii, Pseudomonas aeruginosa*, and *Klebsiella pneumoniae* can develop resistance to nearly all available antibiotics, posing some of the most serious challenges in modern medicine (Baindara et al., 2025). Due to the lack of effective alternative antibiotics, polymyxins are often used as a last-line therapy against these ‘superbugs’ (Nang et al., 2021). However, polymyxins can cause nephrotoxicity in up to 60% of patients after intravenous administration, which is the major dose-limiting factor limiting their clinical use (Azad et al., 2019; Wang et al., 2022). Thus, there is an urgent need for safer, new-generation lipopeptide antibiotics (Slingerland and Martin, 2024).

It has been demonstrated that polymyxin-induced nephrotoxicity is associated with extensive reabsorption into renal proximal tubular cells (Azad et al., 2019). The intracellular concentration of polymyxins in renal proximal tubular cells can be 5000-fold higher than the extracellular concentration, which consequently leads to oxidative stress, autophagy, cell cycle arrest, and apoptosis (Azad et al., 2019). Therefore, the extensive reabsorption of polymyxins into renal proximal tubular cells is the very first step in nephrotoxicity.

Human solute carrier transporters (SLCs) are the primary influx transporters responsible for the cellular uptake of many drugs, including antibiotics (Lu et al., 2018; Zhou et al., 2017). There are over 300 SLC isoforms, among which the SLCO, SLC22A, and SLC15A subfamilies are key in mediating drug transport across the cell membrane (Chan et al., 2016b; Luo et al., 2023). Our previous study revealed that the cellular uptake of polymyxins is mediated through hPepT2 in the kidney (Lu et al., 2016), which is encoded by a key isoform of the SLC15A subfamily, human oligopeptide transporter 2 (*SLC15A2*) (Luo et al., 2023). This transporter protein is highly expressed in renal tubular cells, mediating the uptake of polymyxins, with a comparable kinetic constant (*K*_m_, reflecting transporter-substrate binding affinity) to its prototype substrates (Luo et al., 2023; Lu et al., 2016; Ocheltree et al., 2004). It is one of the proton-driven symporters, using the inwardly directed proton electrochemical gradient to drive the concentrative uptake of substrates across the cell membrane (Smith et al., 2013).

Substrates of hPepT2 include a range of di- or tri-peptides and peptide-like drugs, such as angiotensin-converting enzyme inhibitors (Zhao and Lu, 2015). For instance, glycylsarcosine (Gly-Sar) is a synthetic dipeptide widely used as a model substrate for hPepT2, exhibiting a moderate binding affinity with a reported *K_m_* value of approximately 100 µM (Xiang et al., 2006). Fluorescent peptide probes, such as 7-amino-4-methylcoumarin-3-acetic acid, have also been employed to evaluate hPepT2 function, with a reported *K_m_* value of approximately 780 µM (Alghamdi et al., 2018). Several β-lactam antibiotics, including cefadroxil, have been identified as substrates of hPepT2, while antiviral agents, such as valacyclovir, can also be transported by this transporter (Shen et al., 2005; Ganapathy et al., 1998). Although many β-lactam antibiotics are substrates of hPepT2, some compounds, including cloxacillin and cefadroxil, have been reported to act as competitive inhibitors of hPepT2 (Newstead et al., 2024). However, it remains unclear how hPepT2 recognizes lipopeptide antibiotics, particularly polymyxins, and this knowledge is critical for elucidating their interaction with polymyxins and its contribution to polymyxin-induced nephrotoxicity.

Here, we integrated computational and experimental approaches to elucidate the interaction between hPepT2 and polymyxins. Importantly, we conducted a proof-of-concept chemical biology study by designing novel lipopeptides and evaluating their cellular uptake, nephrotoxicity, and antibacterial activity. This study establishes the first structure-interaction relationship (SIR) model for polymyxins and hPepT2, laying the foundation for the rational drug design and discovery of novel lipopeptide antibiotics.

## Results

### MD prediction of the structural model of outward-facing hPepT2

**Figure 1.**
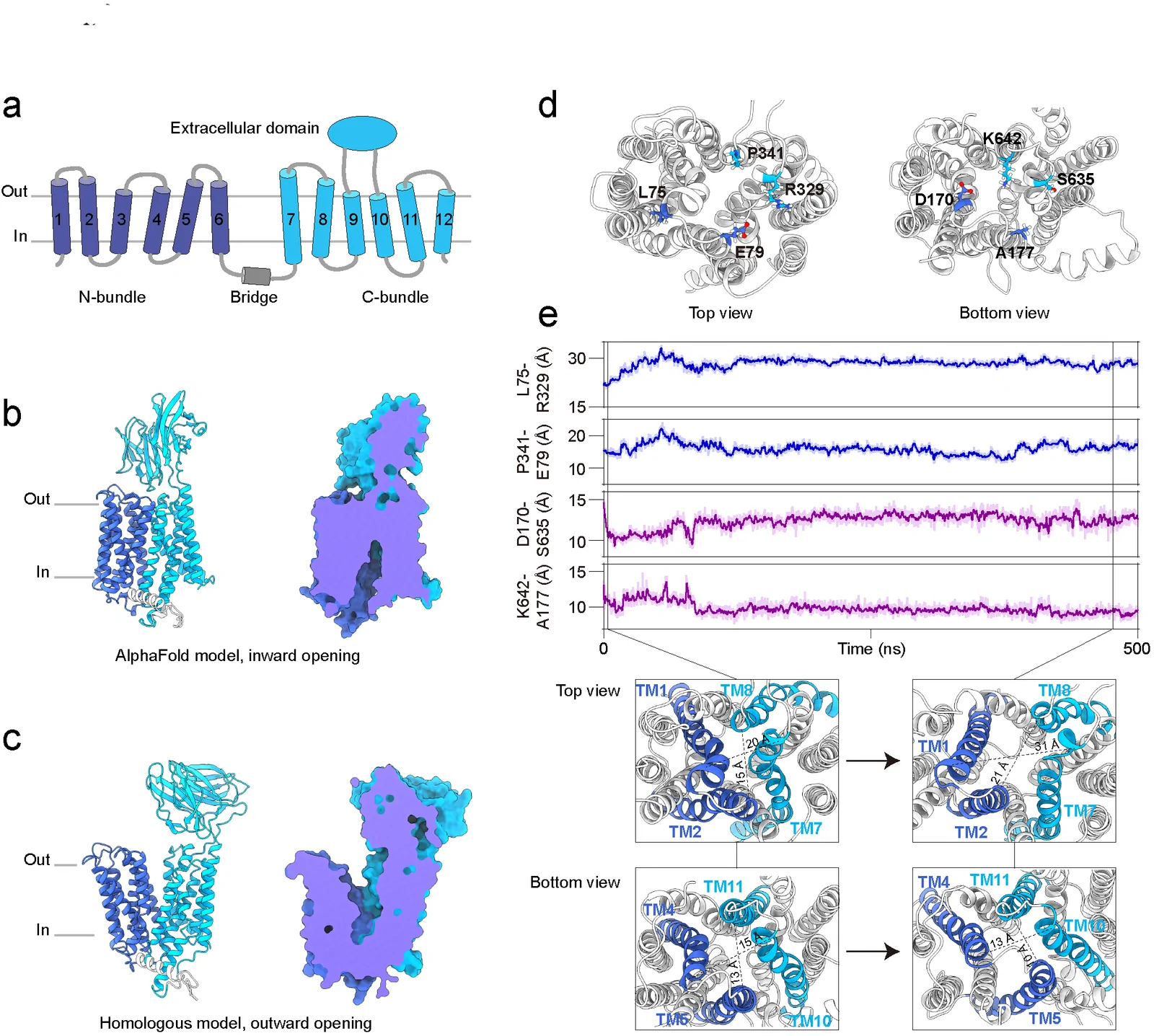
Structural conformations of human oligopeptide transporter 2 (hPepT2). (**a**) The structural topology of hPepT2. (**b**) The structural model of hPepT2 built with the AlphaFold approach. (**c**) The structural model of hPepT2 built with the homologous modeling method. The three-dimensional structure of rat PepT2 (PDB code: 7NQK) is used as the structural template. (**d**) The residues used to define the geometry of the channel of hPepT2. (**e**) The distances between the selected residues and representative structural snapshots are analyzed based on molecular dynamics (MD) simulations. Figure 1—source data 1.The time-evolved distances between key hPepT2 residues derived from MD simulations.

We developed the three-dimensional structure of hPepT2 using AlphaFold2, as the outward structure of hPepT2 (Figure 1a) has not been experimentally solved. The putative overall structure adopted an inward-open conformation (Figure 1b), which prevented the exploration of the initial capture of polymyxins from the extracellular side of the membrane. Parker et al*.* demonstrated the cryo-electron microscopy (cryo-EM) structure of rat PepT2 with an outward-open conformation (Parker et al., 2021). As rat PepT2 shares 83% sequence similarity with hPepT2, it served as a suitable template for modeling the hPepT2 structure through homology modeling. Upon remodeling, the resultant structure of hPepT2 adopted an outward-open conformation (Figure 1c). Several transmembrane domains (TMs) are involved in the formation of the solvent-accessible cavity for polymyxin translocation, including TM1, TM2, TM5, TM7, TM8, and TM10. The gate residue pairs (L75-R329, P341-E79, D170-S635, and K642-A177) marking the conformations were clearly identified in the solvent-accessible regions on both the extracellular and intracellular surfaces (Figure 1d). During the 500-ns all-atom MD simulations with the inward-facing conformation as an initial configuration, the distances between the extracellular gate residue pairs L75-R329 and P341-E79 increased from 20 Å to 31 Å and from 15 Å to 21 Å, respectively. Meanwhile, the distances between the intracellular gate residue pairs D170-S635 and K642-A177 decreased from 15 Å to 13 Å and from 13 Å to 10 Å, respectively (Figure 1e). Notably, the transition from the inward conformation to the outward conformation uncovered by MD simulations is consistent with the homology model of rat PepT2 regarding the distance of the extracellular and intracellular gates (Parker et al., 2021). This observation indicates the accuracy of the proposed hPepT2 model.

### Polymyxin B binds to the lateral opening site of hPepT2 to enter the transport pathway

**Figure 2.**
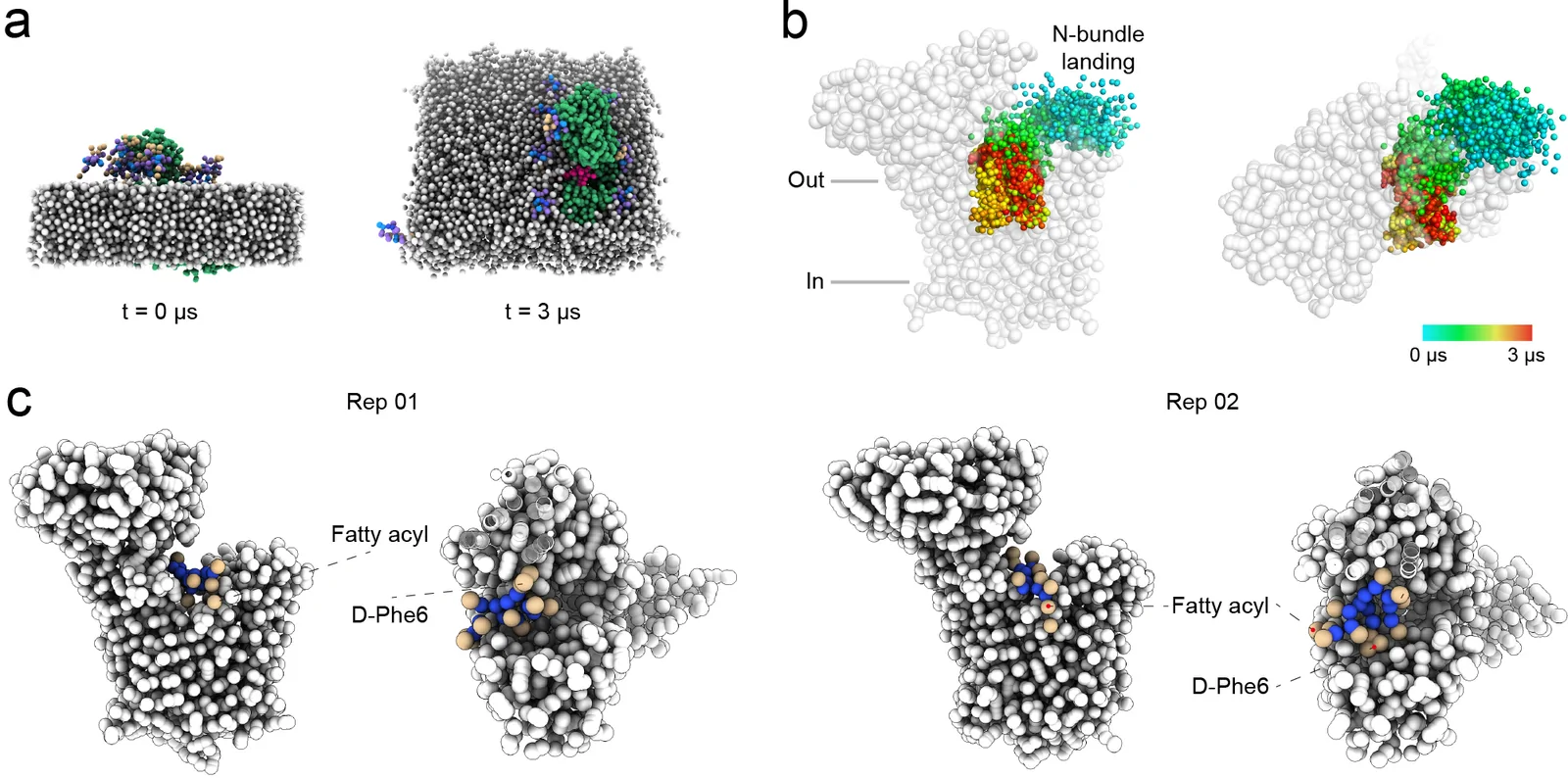
Spontaneous interactions of polymyxin B with human oligopeptide transporter 2 (hPepT2). (**a**) The interaction pathway of polymyxin B with hPepT2. The spatial locations of polymyxin B molecules are represented with colored spheres. The color spectrum from cyan to red indicates the simulation continuity from 0 to 3 μs. (**b**) The final state of two coarse-grained simulation replicates. hPepT2 is shown in gray spheres, and polymyxin molecules are shown in brown and blue spheres. The fatty acyl group and D-Phe6 of polymyxin B are labeled. (**c**) The equilibrated conformation of the hPepT2-polymyxin complex according to the proposed pathway. The polymyxin B molecule is shown in brown and blue spheres with its D-Phe6 and *N*-terminal fatty acyl group labeled.

**Figure 3.**
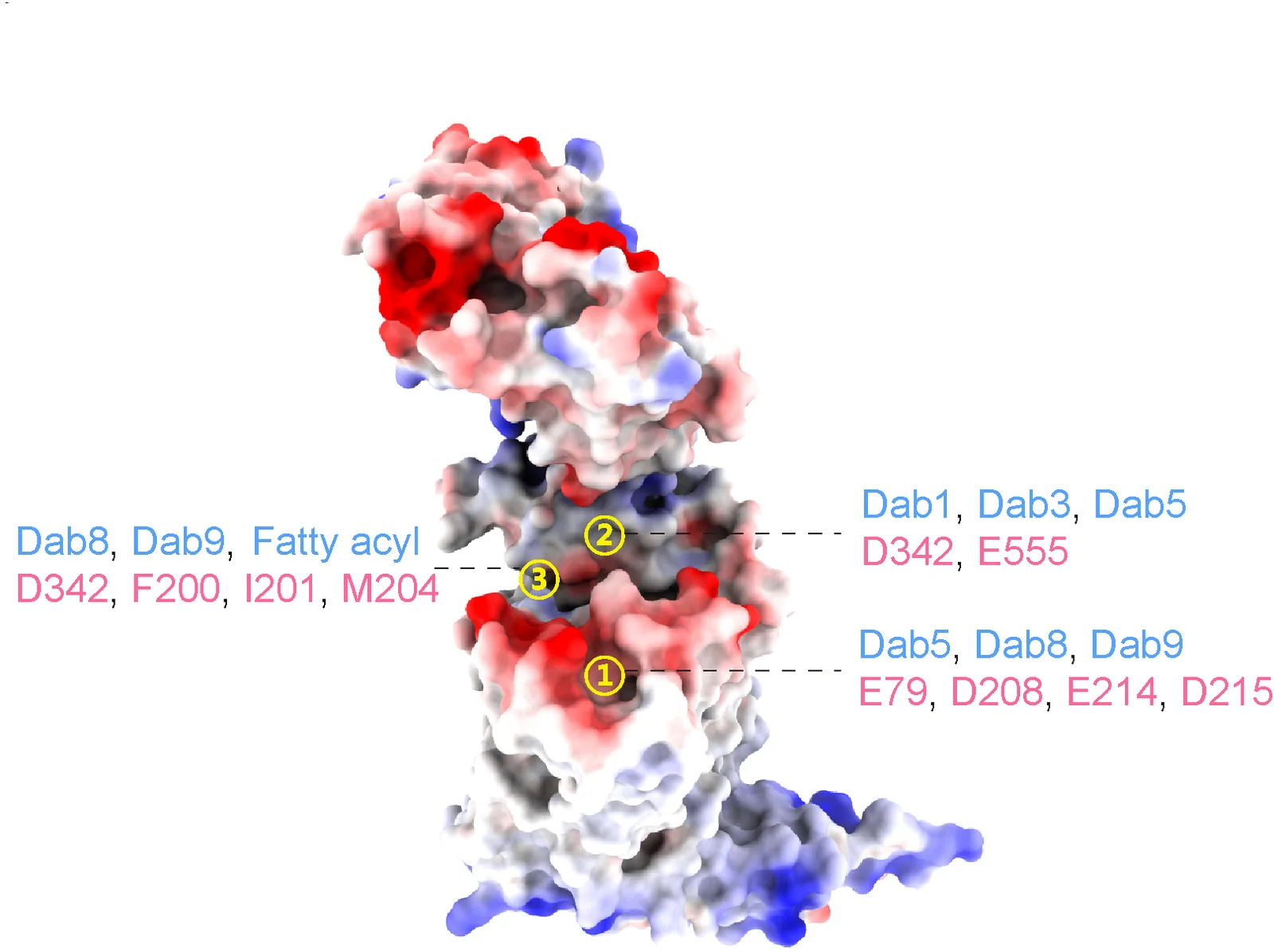
Key structural moieties and residues involved in the interaction of polymyxin B with human oligopeptide transporter 2 (hPepT2). The surface potential of hPepT2 is shown with red color indicating negative potential and blue color indicating positive potential.

To further explore how hPepT2 recognizes polymyxin molecules, we examined their interaction through long-timescale coarse-grained MD simulations. Although ten polymyxin B molecules were introduced into the simulation system, only one polymyxin molecule was observed to bind to the entrance of the hPepT2 binding pocket, while the other polymyxin molecules attached to the membrane lipids (Figure 2a). Importantly, we discovered a distinct pathway by which polymyxins reach the extracellular lateral opening gate of hPepT2, as revealed by the simulation replicates (Figure 2b and c), with distinct equilibrated binding conformations (Figure 2c). Our MD simulations predicted a possible transport pathway for polymyxins via hPepT2. Initially, polymyxin B attached to the plateau of the TM1-TM6 helix bundle, mainly driven by electrostatic interactions between the Dab5, Dab8, and Dab9 residues of polymyxin B and the E79, D208, E214, and D215 residues of hPepT2 (Figure 3). Subsequently, polymyxin B translocated to the junction between the TM7-TM12 helix bundle and the extracellular domains (ECDs) of hPepT2, where the Dab1, Dab3, and Dab5 residues of polymyxin B electrostatically interacted with the D342 and E555 residues of hPepT2. Polymyxin B then stabilized at the lateral opening gate of hPepT2 (i.e. lateral entry site), where the Dab8 and Dab9 moieties of polymyxin B electrostatically interacted with the D342 and E555 residues of hPepT2. Moreover, the hydrophobic atoms in Dab8 and Dab9 interacted with the F200, I201, and M204 residues of hPepT2, while the fatty acyl group of polymyxin B bound to the hydrocarbon tails of the phospholipid bilayer.

**Figure 4.**
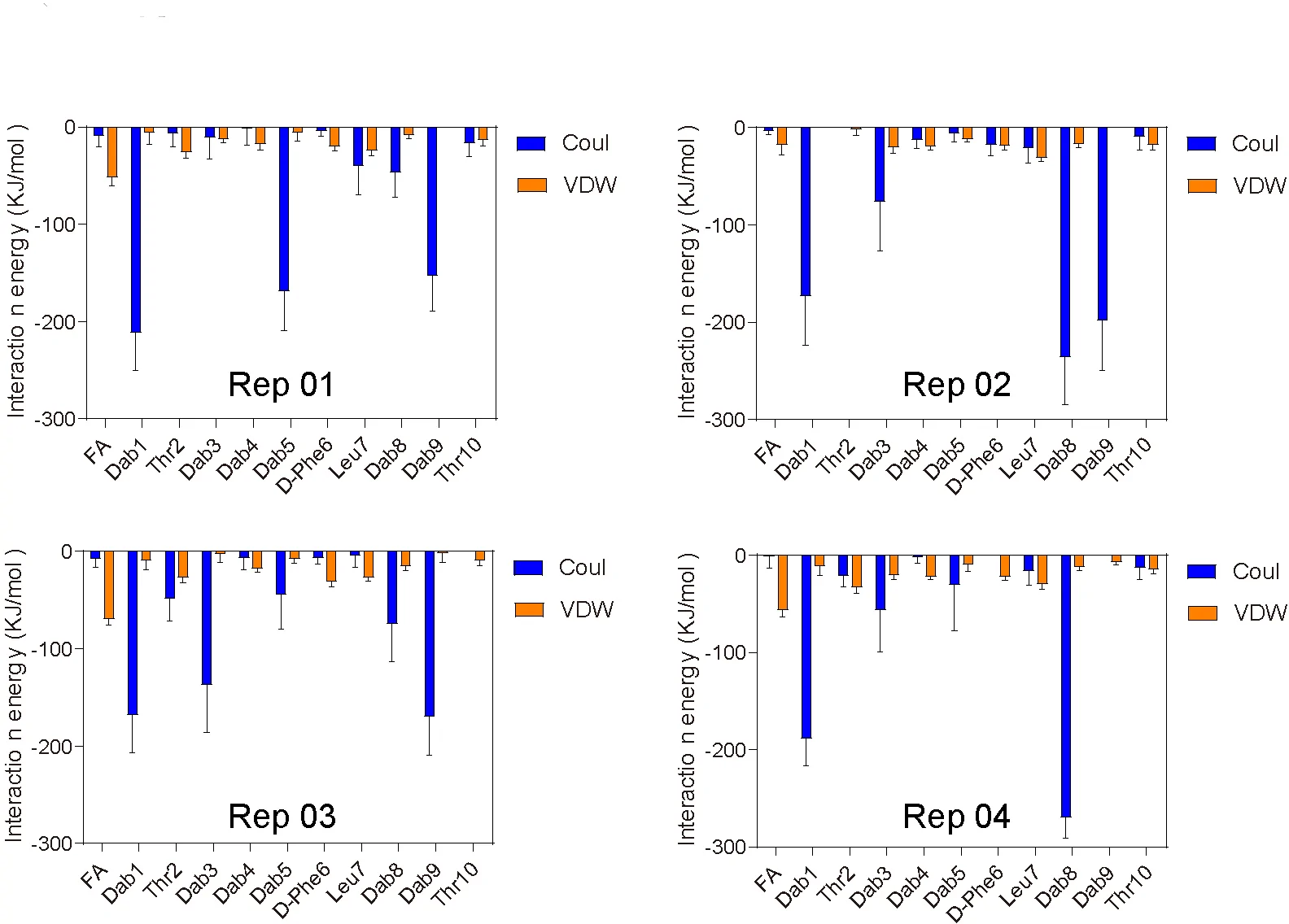
Interaction energy between human oligopeptide transporter 2 (hPepT2) and each residue of polymyxin B. The interaction energy was calculated using four molecular dynamics (MD) simulation replicates. **Note:** Coul: electrostatic interaction; VDW: hydrophobic interaction. Figure 4—source data 1.The interaction energy between each polymyxin residue and human oligopeptide transporter 2 (hPepT2) derived from molecular dynamics (MD) simulation replicas.

To examine the detailed interactions between hPepT2 and polymyxin B, we performed all-atom MD simulations of the hPepT2-polymyxin B complex. During system preparation, the polymyxin B molecule was placed in four different poses within the complex to enhance the sampling of binding dynamics. The results revealed that Dab1, Dab8, and Dab9 form strong electrostatic interactions with hPepT2, with interaction energies ranging from –180 to –260 kJ/mol (Figure 4), while Dab3 and Dab5 may also contribute significantly. Together with observations from the coarse-grained simulations, these results indicate that the positively charged Dab residues are key determinants of their binding affinity to hPepT2.

### Functional mutagenesis elaborates on the predicted transport pathway

At physiological pH, there are five positively charged residues (i.e. Dab residues) on the polymyxin molecule, and we assumed that the key negatively charged residues of hPepT2 identified by the MD simulations (i.e. E79, D208, E214, D215, D317, D342, E555, and E622) may play crucial roles in the recognition of and interactions with polymyxins. We employed functional mutagenesis to confirm their roles in the uptake of polymyxins, as predicted by our MD studies. Through the commonly used alanine-scanning mutagenesis approach, we constructed the corresponding mutants of hPepT2. Notably, alanine substitution not only impacted the side-chain length of the proposed residues but also altered their charges from negative to neutral. It is postulated that such a drastic change in the residues of hPepT2 may substantially affect their interactions with polymyxins.

**Figure 5.**
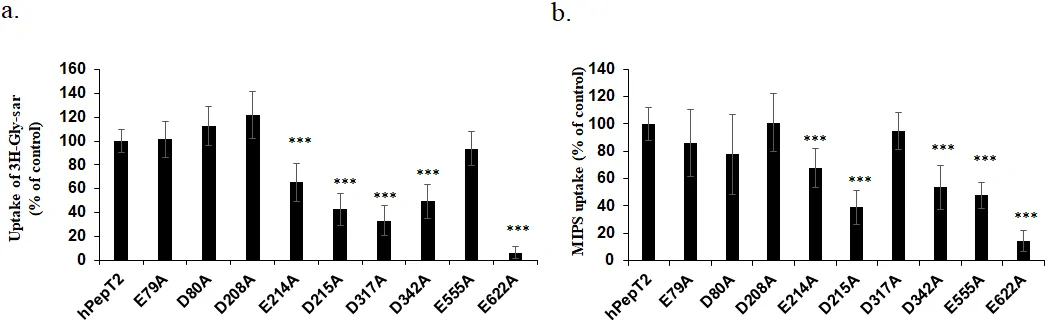
Uptake of ^3^H-Glycosarcosine and polymyxin B fluorescence probe MIPS-9541 by human oligopeptide transporter 2 (hPepT2) and its mutants. HEK293 cells were transfected with hPepT2 and its mutant constructs. (**a**) Uptake of [^3^H]-Gly-Sar (5 µM) by hPepT2 or its mutants. (**b**) Uptake of MIPS-9541 (10 µM) by hPepT2 or its mutants. Data are presented as mean± SD. All experiments were performed in triplicate independently. ***p<0.001 *vs*. control by Welch’s *t*-test. Figure 5—source data 1.The original data used to derive the substrate uptake of human oligopeptide transporter 2 (hPepT2) and its mutants in Figure 5.

**Figure 6.**
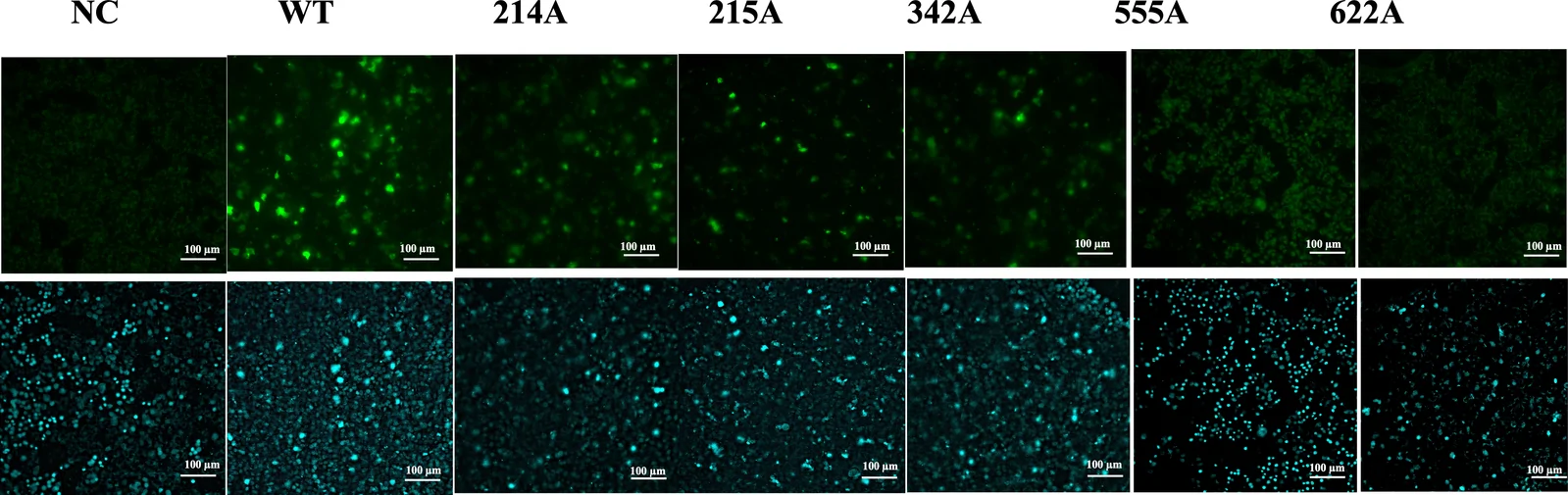
Uptake of the polymyxin B fluorescence probe MIPS-9541 in HEK293 cells overexpressing human oligopeptide transporter 2 (hPepT2) or its mutants assessed by fluorescence imaging. The fluorescence signaling of MIPS-9541 (green) was shown in the upper panel; the nuclei staining (blue) was shown in the bottom panel. Data are presented as mean± SD. All experiments were performed in triplicate independently. Note: WT: wild-type; NC: negative control. Figure 6—source data 1.The original fluorescence images taken for human oligopeptide transporter 2 (hPepT2), its mutants and vector-expressing HEK293 cells.The upper panels are images taken for MIPS-9541; the bottom panels are images taken for nuclei staining. NC: vector-transfected negative control.

The transport activity of these hPepT2 mutants was evaluated by assessing the uptake of H^3^-Gly-Sar (a typical substrate of hPepT2) (Figure 5a; Lu et al., 2016) as well as the fluorescent polymyxin probe MIPS-9541 (Figure 5b). The fluorescence images were also captured as a qualitative measurement of the overexpressing cells exposed to our dansyl-fluorescent polymyxin probe MIPS-9541, which indicated consistent results (Figure 6). Our data showed that E79A, D80A, and D208A mutants maintained transport activity in the uptake of both substrates (Figure 5). In contrast, E214A, D215A, D342A, and E622A mutants had impaired function in the transport of either substrate. The uptake of Gly-Sar was reduced to 65.4±15.8%, 42.7±13.2%, 49.4±14.2% and 6.4±4.7% of the wild-type, respectively (Figure 5a). In the case of MIPS-9541, the cellular uptake was decreased to 67.6±14.2%, 38.9±12.2%, 53.7±16.2%, and 14.3±8.0% of the wild-type, respectively. Interestingly, D317A and E555A showed differential activity in the uptake of these substrates (33.1±12.7% of the control for D317A and 93.6±14.4% of the control for E555A in the uptake of Gly-Sar; 94.9±13.3% of the control for D317A and 47.7±16.2% of the control for E555A in the uptake of MIPS-9541), suggesting the distinct roles of both residues in interactions with different substrates.

Subsequently, kinetic analysis was conducted to characterize those hPepT2 mutants with impaired activity in the uptake of MIPS-9541 (i.e. E214A, D215A, D342A, and E555A). Notably, the E622A mutant was not included in this analysis due to its critically low transporter function. As shown in Table 1, the D215A mutant exhibited an approximately twofold increase in both K_m_ (p=0.033, 95% confidence interval of 7.5–91.5 µM) and V_max_ (p=0.017, 95% confidence interval of 1510–7206 pmol/(µg/4 min)). It was also evident that the D342A mutant showed a mildly increased K_m_ (66.9±13.2 µM of D342A *vs*. 47.1±11.9 µM of the wild-type, p=0.13, 95% confidence interval of –9.1–48.7 µM) without altering its V_max_. The V_max_ values of E214A and E555A were significantly reduced (2332±24.2 pmol/(µg/4 min) of E214A, p=0.036, and 2376±320.8 pmol/(µg/4 min) of E555A, p=0.022, *vs* 4252±652.9 pmol/(µg/4 min) of the wild-type). However, the K_m_ values of E214A and E555A mutants were moderately decreased compared to those of the wild-type (24.4±4.5 µM of E214A, p=0.07 and 26.7±7.0 µM of E555A, p=0.08 *vs* 47.1±11.9 µM of the wild-type).

**Table 1.** Transporter kinetic parameters of MIPS-9541 uptake by human oligopeptide transporter 2 (hPepT2) and its mutants. Table 1—source data 1.The original data used to derive the kinetic parameters of human oligopeptide transporter 2 (hPepT2) mutants in the uptake of MIPS-9541.

Construct	K_m_ (µM)	V_max_ (pmol/(µg/4 min))
hPepT2	47.1±11.9	4252±652.9
E214A	24.4±4.48	2332±24.2*
***D215A***	96.6±20.9*	8610±1363*
D342A	66.9±13.2	4396±583.1
E555A	26.7±6.99	2376±320.8*

^*^p<0.05 vs. control by Welch’s t-test.

**Figure 7.**
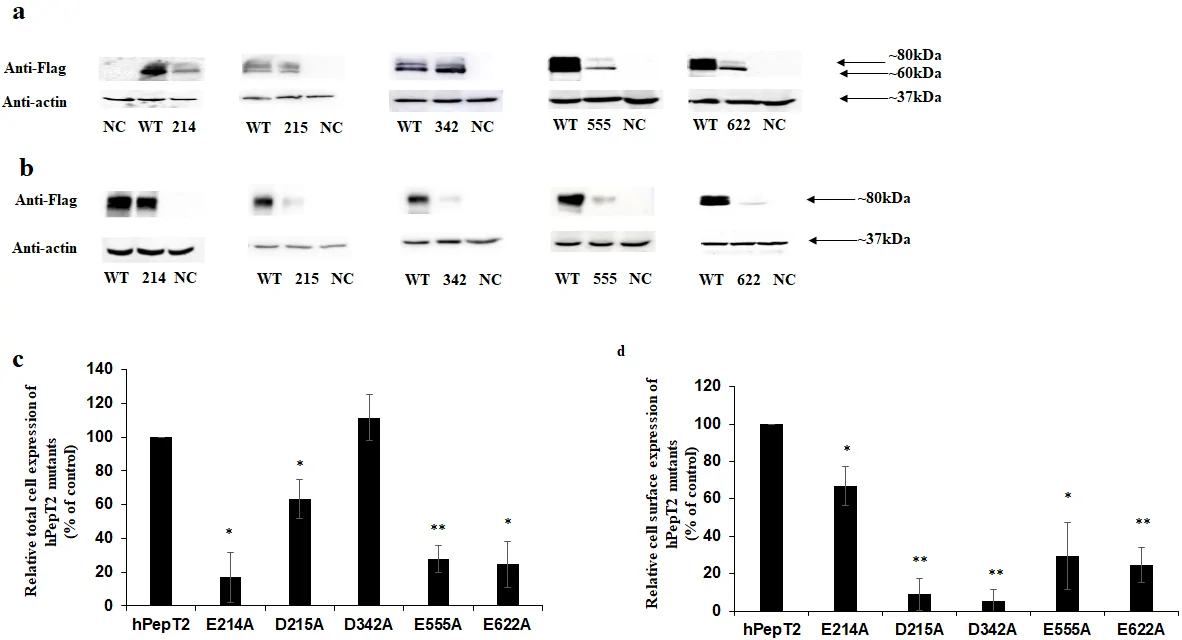
Total cell and cell surface expression of human oligopeptide transporter 2 (hPepT2)-c-Flag and its mutants. HEK293 cells were transfected with hPepT2-c-Flag and its mutant constructs. (**a**) Cells were lysed and subjected to SDS-electrophoresis. Immunoblots were probed with an anti-Flag antibody followed by probing with an anti-actin antibody as a loading control. Representative images of each hPepT2 mutant are shown. (**b**) Cell surface proteins were labeled with NHS-ss-Biotin and then pulled down with Streptavidin-agarose beads. Biotin-labeled surface protein samples were subjected to SDS-electrophoresis, and the immunoblots were probed with anti-Flag antibody. Equal portions of the supernatant samples were separated by SDS-PAGE, and the immunoblots were incubated with anti-actin antibody. Representative images of each hPepT2 mutant are shown. (**c**) Densitometry analysis of the relative total cell expression of hPepT2 mutants (ratios of Flag/actin). (**d**) Densitometry analysis of the relative cell surface expression of hPepT2 mutants (ratios of Flag/actin). Data are presented as % of the hPepT2 wild-type control (mean± SD). Experiments were repeated on three occasions. ^*^p<0.05; ^**^p<0.01 *vs.* wild-type control by unpaired *t*-test. Note: WT: wild-type; NC: vector-transfected negative control. Figure 7—source data 1.The original files of the full raw uncropped, unedited gels, or blots.The numbers 214, 215, 342, 555, and 622 indicate the positions of the respective mutations in human oligopeptide transporter 2 (hPepT2). Figure 7—source data 2.Figures with the uncropped gels or blots with the relevant bands clearly labeled.The upper panels show blots probed with an anti-Flag antibody to detect human oligopeptide transporter 2 (hPepT2) and its mutants, while the lower panels show blots probed with an anti-actin antibody as loading controls. The left panels show blots of total cell lysates, whereas the right panels show blots from the cell-surface biotinylation assay. WT, wild-type; NC, vector-transfected negative control. The numbers 214, 215, 342, 555, and 622 indicate the positions of the respective mutations in hPepT2.

Notably, a change in V_max_ value may be due to altered transporter protein expression and/or transporter turnover rate. Thus, the total cell and plasma membrane expression of hPepT2 and its mutants were investigated to further examine the molecular mechanism underpinning the functional alterations of these mutants. As shown in Figure 7, E214A, D215A, E555A, and E622A significantly reduced total cell expression and cell surface expression. In the case of D342A, it retained transporter protein expression in the whole cell but had a markedly impaired cell surface expression.

### Chemical biology of polymyxin interaction with hPepT2

**Figure 8.**
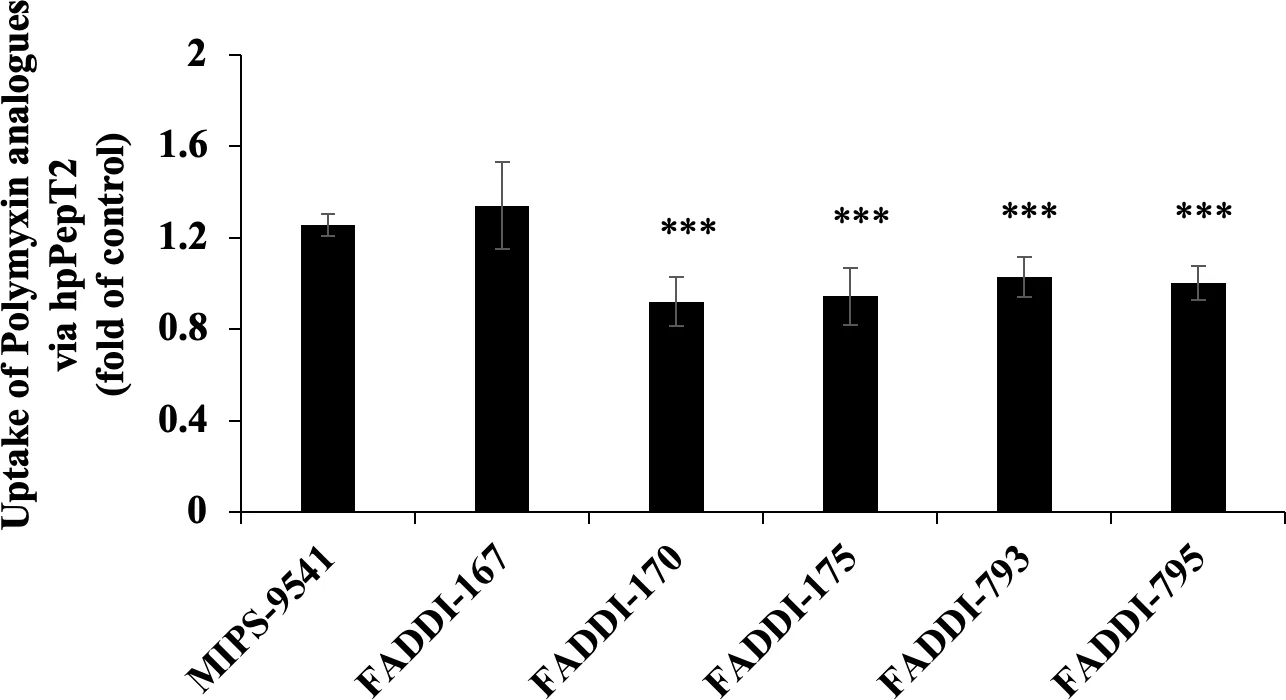
Uptake of the polymyxin B fluorescence probe MIPS-9541 and polymyxin analogs by human oligopeptide transporter 2 (hPepT2). The data are expressed as the fold change in the uptake via hPepT2 *vs.* that of the vector-transfected control. Data are presented as mean± SD. All experiments were performed in triplicate independently. ***p<0.001 vs. MIPS-9541 control by Welch’s *t*-test. Figure 8—source data 1.The original data of human oligopeptide transporter 2 (hPepT2)-mediated uptake of polymyxin B1 and its derivatives.

As described above, the Dab1, Dab3, Dab5, Dab8, and Dab9 residues of polymyxin B were predicted to be involved in the interaction with hPepT2. To test this hypothesis, we designed and synthesized five polymyxin B analogs with alanine substitutions at these Dab positions. We performed a transport uptake assay to evaluate their interactions with hPepT2. As shown in Figure 8, the lipopeptide analogs FADDI-170, FADDI-175, FADDI-793, and FADDI-795, but not FADDI-167, showed reduced uptake in hPepT2-expressing cells, indicating that Dab1, Dab3, Dab5, and Dab9, but not Dab8, are likely involved in the interaction with hPepT2. Therefore, these four lipopeptide analogs may have less intracellular accumulation and reduced nephrotoxicity.

### Pharmacological evaluations of lead polymyxin analogs

We first assessed the minimum inhibitory concentrations (MICs, the lowest concentration that inhibits visible growth of a microorganism after overnight culture) of the four lipopeptide analogs predicted to have disrupted interactions with hPepT2. As shown in Table 2, FADDI-170 showed significantly reduced activity against FADDI-PA025, FADDI-EC006, FADDI-EC003, FADDI-EC001, FADDI-AB034, as well as *A. baumannii* ATCC 19606 and ATCC 17978 (MICs increased ≥16 fold, compared to polymyxin B). FADDI-175 displayed low activity against all the tested strains (MICs ≥32 µg/mL), which was not further considered for subsequent testing. FADDI-793 was less active than polymyxin B against *A. baumannii* ATCC 19606, ATCC 17978, FADDI-AB034, FADDI-EC006, and FADDI-EC003. FADDI-795 was the most active among them, showing comparable MICs against these bacterial strains except for FADDI-PA025 when compared to polymyxin B. Interestingly, FADDI-793 and FADDI-795 were more active than polymyxin B against *K. pneumoniae* ATCC 13883. We then employed the mouse model established in our laboratory (Roberts et al., 2022) to examine the nephrotoxicity of the four antibacterial polymyxin analogs. Interestingly, FADDI-795 showed no observable nephrotoxicity, while the other three analogs possessed mild kidney toxicity, comparable to that of polymyxin B (Table 2).

**Table 2.** Chemical modifications, minimum inhibitory concentrations, and nephrotoxicity of polymyxin B (PMB) and its analogs. Table 2—source data 1.The original data of minimum inhibitory concentrations and nephrotoxicity of polymyxin B and its analogs.

Lipopeptide	Dab → Ala modification position	Minimum inhibitory concentration (µg/mL)											Max Kidney SQS
		*P. aeruginosa* ATCC 27853	*P. aeruginosa* FADDI-PA022	*P. aeruginosa* FADDI-PA025	*A. baumannii* ATCC 19606	*A. baumannii* FADDI-AB034	*A. baumannii* ATCC 17978	*K. pneumoniae* ATCC 13883	*K. pneumoniae* FADDI-KP032	*E. cloacae* FADDI-EC006	*E. cloacae* FADDI-EC001	*E. cloacae* FADDI-EC003	
PMB		1	1	2	1	0.25	0.5	16	0.5	0.5	0.5	<0.125	+1
FADDI-170	9	2	4	32	>32	>32	32	>32	4	8	8	8	+1
FADDI-175	5	>32	>32	>32	>32	>32	>32	>32	>32	>32	32	32	N.D.
FADDI-793	1	2	4	8	>32	>32	16	4	4	8	2	4	+1
FADDI-795	3	4	8	32	8	4	4	4	1	0.5	1	0.5	0

Polymyxin B was chemically modified at the indicated position by replacing Dab with Ala residue. Lipopeptide was administered to mice via intraperitoneal injection at a dose of 8 mg/kg every 2 hr for 6 doses in 1 day (a total dose of 48 mg/kg/day, n = 3). The kidney histology damage score was determined in the form of semi-quantitative score (SQS): SQS=0, no significant kidney damage; SQS=+1, mild kidney damage; N.D. not determined (Roberts et al., 2015).

Overall, the transporter uptake study and pharmacological assessments of these polymyxin analogs provide critical mechanistic data on the interaction and transport of polymyxins by hPepT2. Importantly, our integrated computational and cell biology approach highlighted the feasibility of reducing polymyxin nephrotoxicity by disrupting their interactions with hPepT2. Moreover, hPepT2 is a viable target for designing novel polymyxin-like lipopeptide antibiotics with reduced nephrotoxicity.

## Discussion

Life-threatening MDR bacterial infections rank among the world’s most pressing health threats (Li et al., 2006), yet the discovery of antibiotics with novel mechanisms of action has stalled for decades. Polymyxins remain a last-line therapy for MDR Gram-negative bacterial pathogens, particularly in low- to middle-income countries; however, their clinical use has been significantly limited due to nephrotoxicity (Nang et al., 2021; Azad et al., 2019).

Our previous study demonstrated that polymyxins are substrates of hPepT2, which mediates the substantial reabsorption of polymyxins in renal proximal tubular cells, leading to nephrotoxicity (Lu et al., 2016). However, little is known about the interaction between polymyxin molecules and hPepT2. Notably, previous studies have reported that megalin mediates the cellular transport of polymyxins in the kidney via a receptor-mediated endocytosis mechanism (Azad et al., 2019; Abdelraouf et al., 2014). Receptor-mediated endocytosis is closely linked to changes in signaling pathways; thus, disrupting the interaction between polymyxins and megalin may lead to potential signaling consequences and complicate treatment outcomes. In contrast, transporter-mediated uptake does not involve such signaling changes, making manipulation of polymyxin-hPepT2 interactions a more favorable strategy for mitigating polymyxin-associated nephrotoxicity. Furthermore, our previous study demonstrated a direct structural interaction of polymyxins with the membrane of human kidney proximal tubular cells (Jiang et al., 2020), showing that polymyxin B inserts its fatty acyl tail into the hydrophobic region of the cell membrane. The stereochemistry at position 3 and the hydrophobicity at positions 6 and 7 of polymyxin B were found to be critical for this interaction. These findings provide important insights into how polymyxins alter membrane dynamics and lipid organization in kidney cells, contributing to a better understanding of polymyxin-induced nephrotoxicity.

The current study developed the first SIR model of polymyxins with hPepT2, expediting the discovery of novel safer lipopeptide antibiotics. We first employed MD simulations to predict the binding of polymyxins to hPepT2 (Figures 1 and 2) and identified several critical amino acid residues of hPepT2 in the recognition of polymyxins. An outward-facing model of hPepT2 was first constructed, indicating the access of polymyxin molecules to the lateral opening gate of hPepT2 (Figure 3). Limited by the sampling capability of MD simulations, the complete translocation pathway of polymyxin B through hPepT2 was not observed. The distinct conformation of polymyxin B indicates that it is situated in the central binding cavity of hPepT2. To further explore the conformational change of polymyxin molecules required for hPepT2 transport, all-atom MD simulations were conducted by varying the orientations of polymyxin B. Our results revealed that this conformational transition involved a notable flip of polymyxin B, which enhanced the interaction between its fatty acyl chain and the deeper regions of the predicted hPepT2 binding pocket. Notably, the subsequent transporter mutagenesis and molecular characterizations of hPepT2 explored our proposed SIR model. Our integrated computational and experimental findings provide a proof-of-concept that disrupting the interaction between polymyxin and hPepT2 is a novel strategy to reduce their nephrotoxicity.

As polymyxins are cationic at physiological pH, we strategically selected the negatively charged amino acids of hPepT2 (i.e. aspartic acid and glutamic acid) for further investigation. The significantly reduced uptake of polymyxin B by hPepT2 mutants E214A, D215A, D342A, E555A, and E622A demonstrated the critical role of these negatively charged residues in transporting polymyxins (Figures 5 and 6). Moreover, the kinetic results of these transporter mutants elucidated the mechanism underpinning such functional impairment (Table 1). As both transporter cell surface expression and turnover rate account for the maximum velocity V_max_ of a transporter protein (Luo et al., 2023), it was critical to evaluate the protein expression of these transporter mutants in total cell lysates and at the cell surface (Figure 7). Both the kinetic analysis and protein expression profiles (Table 3) showed different mechanisms of impaired polymyxin uptake by hPepT2. For example, it is highly likely that E214A reduced V_max_ due to reduced total cell and membrane protein expression. D215A reduced polymyxin uptake mainly due to impaired binding affinity to polymyxins (i.e. increased K_m_). The transporter turnover rate of D215A might increase to compensate for the reduced cell surface expression; thus, its V_max_ was overall increased. Similarly, the decreased polymyxin uptake mediated via D342A may be primarily attributed to its reduced polymyxin binding affinity. Its decreased membrane protein expression and likely elevated transporter turnover rate may have resulted in an unchanged V_max_. In contrast, the dysfunction of E555A may be mainly due to its reduced V_max_, likely resulting from a reduced total cell and membrane protein expression, although its binding affinity to polymyxins is slightly increased (i.e. decreased K_m_). The kinetic parameters of E622A were unable to be determined due to its low polymyxin uptake. Our data also indicated that its low protein expression in total cells and at the cell surface may contribute to its impaired transport activity in polymyxin uptake. Notably, reduced total cellular expression of membrane transporters may result from impaired protein stability (Chan et al., 2015a). In addition, cell surface transporter proteins undergo constitutive subcellular trafficking processes, including internalization, recycling, and membrane targeting (Chan et al., 2016a). Therefore, decreased cell surface expression of transporter proteins may arise from reduced total cellular expression and/or disrupted subcellular trafficking (Chan et al., 2015a). Our findings suggest that E214, D215, E555, and E622 residues may play important roles in maintaining hPepT2 protein stability, whereas substitution of these residues with alanine may influence the subcellular trafficking of hPepT2. Nevertheless, the detailed molecular mechanisms underlying the changes in hPepT2 protein expression associated with these residues warrant further investigation but are beyond the scope of the current study.

**Table 3.** The kinetic parameters and protein expression profiles of human oligopeptide transporter 2 (hPepT2) mutant constructs.

hPepT2 mutant	K_m_	V_max_	Total cell expression	Cell surface expression
E214A	↓	↓↓	↓↓	↓↓
D215A	↑↑	↑↑	↓↓	↓↓
D342A	↑	*Unchanged*	*Unchanged*	↓↓
E555A	↓	↓↓	↓↓	↓↓
E622A	N.D.	N.D.	↓↓	↓↓

↓: mildly or moderately decreased; ↓↓: significantly decreased; ↑: mildly or moderately increased; ↑↑: significantly increased; N.D.: not determined.

Notably, because some transporter mutants (e.g. D342A) exhibited both increased K_m_ and reduced membrane expression; it is not possible to conclusively determine whether the decreased transporter function results from reduced binding affinity, lower protein expression, or a combination of both. This is a limitation of the current study. Nevertheless, our molecular assay results support the applicability of the bioinformatic predictions, as they allow us to focus the analysis on a limited set of transporter residues most relevant to our SIR model.

To validate the MD predictions, we further investigated the transport function of these mutant transporters through their uptake of the classic hPepT2 substrate Gly-Sar (Figure 5a). The uptake of Gly-Sar was dramatically decreased in the mutants E214A, D215A, D317A, D342A, and E622A, which is consistent with the reduced cell surface expression of E214A, D215A, D342A, and E622A (Figure 7). D317A is exceptional as it showed decreased uptake of Gly-Sar but maintained transport function in the case of polymyxin B, which indicated that the residue D317 may be only important for the uptake of Gly-Sar but not polymyxins. E555A is another exception, which remained functional for the uptake of Gly-Sar but reduced uptake of polymyxins. As E555A had a reduced total cell and cell surface expression, its increased apparent affinity for Gly-Sar may partially compensate for the reduced V_max_. Thus, the binding determinants of polymyxins may not fully overlap with those of Gly-Sar. Therefore, the transport inhibition study was only employed as a positive control initially and not considered in the subsequent evaluation of the antibacterial activity of polymyxin analogs.

According to the predicted topology model of hPepT2 (Luo et al., 2023), E214A and D215A are situated at the ECD between TM5 and TM6. D342A resides at the ECD between TM7 and TM8. E555A is located at the ECD between TM9 and TM10. E622A is situated within TM10. It was reported that the large ECD between TM9 and TM10 interacts with TM1 to maintain transport function (Shen et al., 2022). In our study, we demonstrated that substitution of E555 mildly increased the apparent binding affinity for polymyxin B while reducing V_max_, likely resulting from decreased protein expression. These findings highlight the need for more in-depth mechanistic understanding of hPepT2-mediated peptide transport. Notably, E622A in TM10 has a very low transport function in the uptake of polymyxin B, which is largely due to its low protein expression in the total cell and on the cell surface (Figures 5 and 7). Therefore, this residue appears to be essential for preserving hPepT2 stability. Similar findings have been reported in other SLCs (Chan et al., 2015a; Hong et al., 2010; Hong et al., 2007). According to the protein sequence alignment between hPepT2 and another key isoform of the SLC15A subfamily, hPepT1, E595 of hPepT1 is the equivalent residue to E622 in hPepT2. It has been shown that the alteration of the E595 residue to cysteine substantially impacted transport function but not protein expression (Xu et al., 2009). This finding reflects the differing substrate specificities and transport capacities of hPepT1 and hPepT2 (Luo et al., 2023), highlighting the essential role of hPepT2 in mediating polymyxin transport.

Upon validating the predictions of the SIR model of polymyxins and hPepT2, our study was advanced to employ a chemical biology approach to rationally design and in-house synthesize polymyxin analogs. With the hope of disrupting the interaction between hPepT2 and these analogs, we aimed to discover candidate lipopeptide antibiotics with reduced nephrotoxicity while maintaining similar or better antibacterial activity. Based on the SIR model, the Dab1, Dab3, Dab5, Dab8, and Dab9 residues of polymyxins are likely to be involved in their interaction with hPepT2 (Figure 4). Thus, we synthesized FADDI-167, FADDI-170, FADDI-175, FADDI-793, and FADDI-795 with alanine substitutions at these Dab residues. We first examined the hPepT2-mediated uptake of these polymyxin analogs. Interestingly, we discovered that all analogs except FADDI-167 disrupted the uptake via hPepT2 (Figure 8). This finding indicated that FADDI-170, FADDI-175, FADDI-793, and FADDI-795 possibly impaired their uptake via hPepT2, which may contribute to reduced renal accumulation and nephrotoxicity. Thus, these four analogs were subjected to further pharmacological evaluations. Interestingly, FADDI-795 and FADDI-175 showed the best and least antibacterial activity among them, respectively (Table 4). Finally, we employed our mouse model to evaluate the nephrotoxicity of these lipopeptides. Using histological evaluations, FADDI-795 showed no observable renal toxicity in this mouse model and emerged as the lead compound with comparable antibacterial activity. A limitation of this research is that the nephrotoxicity assessment of new lipopeptides was performed in only a small number of mice in our proof-of-concept study. Nevertheless, the findings indicate that FADDI-795 is a promising candidate that will undergo further pharmacological evaluations to explore its translational potential, such as comprehensive pharmacokinetic studies, renal accumulation assessment, and efficacy tests in relevant infectious disease models.

**Table 4.** Primers used for human oligopeptide transporter 2 (hPepT2) mutagenesis.

Mutant	Forward (5'–3')	Reverse (5'–3')
E79A	atatagatgtggaggtatctgcattccagtgcaggaaatac	gtatttcctgcactggaatgcagatacctccacatctatat
D80A	cctgcactggaatgaagctacctccacatctatatacc	ggtatatagatgtggaggtagcttcattccagtgcagg
D208A	gggtacgactctcctcgacacgttacaaaacc	cccatgctgagaggagctgtgcaatgttttgg
E214A	gccaatgcatagcagtctgctccaaaacattgcacat	atgtgcaatgttttggagcagactgctatgcattggc
D215A	aaaagccaatgcatagcaggcttctccaaaacattgcac	gtgcaatgttttggagaagcctgctatgcattggctttt
D317A	cccgagaaaaccgagtcgtcccaagtgc	gggctcttttggctcagcagggttcacg
D342A	gtgcttcagccggcccagatgcaggttc	gaacctgcatctgggccggctgaagcac
E555A	gtgcactgcagggtatgctcctctttgcacagt	actgtgcaaagaggagcataccctgcagtgcac
E622A	gacagagaacatgaccgccccagctgtaaccag	ctggttacagctggggcggtcatgttctctgtc

Overall, our study developed the first SIR model of polymyxins with hPepT2 by integrating computational, chemical biology, and molecular approaches. Our molecular studies provide the first experimental validation of the predicted polymyxin-hPepT2 binding model. These mechanistic insights are essential for elucidating the mechanism of dose-limiting nephrotoxicity associated with polymyxin treatment. Moreover, our ongoing drug discovery efforts will further refine this SIR model and expedite the discovery and development of novel, safer lipopeptide antibiotics against Gram-negative bacterial infections.

## Materials and methods

**Key resources table:** 

Reagent type (species) or resource	Designation	Source or reference	Identifiers	Additional information
Cell line (*Homo sapiens*)	HEK293T	ATCC	CRL-3216, RRID:CVCL_0063	Human embryonic kidney cells for transient transfection
Strain, strain background (*Pseudomonas aeruginosa)*	*Pseudomonas aeruginosa* (Schroeter) Migula	ATCC	Cat#: 27853	Antibacterial activity test
Strain, strain background (*Pseudomonas aeruginosa)*	FADDI-PA022	Lin et al., 2017b; Lin et al., 2017a		Antibacterial activity test
Strain, strain background (*Pseudomonas aeruginosa)*	FADDI-PA025	Roberts et al., 2022		Antibacterial activity test
Strain, strain background (*Acinetobacter baumannii)*	*Acinetobacter baumannii* Bouvet and Grimont	ATCC	Cat#: 19606	Antibacterial activity test
Strain, strain background (*Pseudomonas aeruginosa)*	FADDI-AB034	Kumar, 2025		Antibacterial activity test
Strain, strain background *(Acinetobacter baumannii)*	*Acinetobacter baumannii* Bouvet and Grimont	ATCC	Cat#: 17978	Antibacterial activity test
Strain, strain background *(Klebsiella pneumoniae* subsp. *Pneumoniae)*	*Klebsiella pneumoniae* subsp. pneumoniae (Schroeter) Trevisan	ATCC	Cat#: 13883	Antibacterial activity test
Strain, strain background *(Klebsiella pneumoniae)*	FADDI-KP032	Deris et al., 2012		Antibacterial activity test
Strain, strain background *(Escherichia coli)*	FADDI-EC006	Roberts et al., 2015		Antibacterial activity test
Strain, strain background *(Enterobacter cloacae)*	FADDI-EC001	Roberts et al., 2015		Antibacterial activity test
Strain, strain background *(Enterobacter cloacae)*	FADDI-EC003	Thombare et al., 2024		Antibacterial activity test
Commercial assay or kit	QuickChange II site-directed mutagenesis kit	Agilent Technologies	Cat#: 200521	Site-directed mutagenesis
Commercial assay or kit	BigDye Terminator v3.1 kit	Thermo Scientific	Cat#: 4337461	DNA sequencing
Commercial assay or kit	Lipofectamine 2000 Reagent	Invitrogen	Cat#: 11668019	Plasmid transfection
Commercial assay or kit	SlowFade Gold Antifade Mountant with DAPI	Invitrogen	Cat#: S36942	Anti-fading for fluorescent probe and nuclear staining
Commercial assay or kit	Streptavidin-Agarose resin	Abcam	Cat#: ab270530	Cell surface biotinylation assay
Commercial assay or kit	Sulfo-NHS-SS-Biotin	Campbell Science	Cat#: BR109	Cell surface biotinylation assay
Commercial assay or kit	Immobilon Western Chemiluminescent HRP substrate	Merck Millipore	Cat#: WBKLS0500	Western blot signal detection
Chemical compound, drug	Glycylsarcosine, [^3^H]	BioScientific Pty. Ltd	Cat#: MT 1545–250	hPepT2 uptake assay substrate
Chemical compound, drug	Glycylsarcosine	Sapphire Biosciences	Cat#: BS-99594C	hPepT2 uptake assay substrate
Chemical compound, drug	Polymyxin B_1_	Sapphire Biosciences	Cat#: 14074	hPepT2 uptake assay substrate
Chemical compound, drug	Polymyxin B sulfate	BetaPharm (Shanghai)	Cat#: C56H100N16O17S	hPepT2 uptake assay substrate and antibiotic used in animal study
Chemical compound, drug	MIPS-9541	Lu et al., 2016; Deris et al., 2014; Yun et al., 2015		hPepT2 uptake assay substrate
Chemical compound, drug	FADDI-167	Roberts et al., 2022; Jiang et al., 2021		Polymyxin analog
Chemical compound, drug	FADDI-170	Roberts et al., 2022; Jiang et al., 2021		Polymyxin analog
Chemical compound, drug	FADDI-175	Roberts et al., 2022; Jiang et al., 2021		Polymyxin analog
Chemical compound, drug	FADDI-793	Roberts et al., 2022; Jiang et al., 2021		Polymyxin analog
Chemical compound, drug	FADDI-795	Roberts et al., 2022; Jiang et al., 2021		Polymyxin analog
Antibody	Flag (DYKDDDDK) rabbit polyclonal antibody	Genesearch	Cat#: 2368, RRID:AB_2217020	Western blot (1:1000 dilution)
Antibody	β-actin mouse monoclonal antibody	Genesearch	Cat#: 4967, RRID:AB_330288	Western blot (1:2000 dilution)
Antibody	Goat anti-rabbit IgG conjugated with HRP 2^nd^ antibody	Sigma-Aldrich	Cat#: A0545, RRID:AB_257896	Western blot (1:5000 dilution)
Antibody	Goat anti-mouse IgG conjugated with HRP 2^nd^ antibody	Sigma-Aldrich	Cat#: A2554, RRID:AB_258008	Western blot (1:10,000 dilution)
Other	Dulbecco’s Modified Eagle Medium (DMEM)	Thermo Scientific	Cat#: 11995073	Cell culture medium
Other	Glutamine	Thermo Scientific	Cat#: 25030081	Cell culture supplement
Other	Fetal bovine serum (FBS)	Gibco	Cat#: 26140–079	Cell culture supplement
Recombinant DNA reagent	hPepT2	Australian Biosearch	Plasmid	PCMV6-XL5-hPepT2
Recombinant DNA reagent	hPepT2-c-Flag	Australian Biosearch	Plasmid	pcDNA3.1+/C-(K)DYK-hPepT2-c-Flag
Software, algorithm	Clustal Omega	University College Dublin	RRID:SCR_001591	Sequence alignment
Software, algorithm	MODELLER	University of California, San Francisco	RRID:SCR_008395	Model prediction
Software, algorithm	AlphaFold2	Google DeepMind	RRID:SCR_025454	Model prediction
Software, algorithm	Martini Maker module	CHARMM-GUI	RRID:SCR_025037	Model construction
Software, algorithm	PyMOL	Schrodinger	Version 3.1.3.1, RRID:SCR_000305	Molecular visualizations, structural figures, and trajectory animations
Software, algorithm	ChimeraX	University of California, San Francisco	Version 1.1, RRID:SCR_015872	Molecular visualizations, structural figures, and trajectory animations
Software, algorithm	AutoDock Vina	The Scripps Research Institute	RRID:SCR_011958	Molecular docking
Software, algorithm	CHARMM-GUI	Lehigh University	RRID:SCR_025037	System preparation
Software, algorithm	GROMACS	University of Groningen	Version 2021.2, RRID:SCR_014565	Molecular dynamic simulations
Software, algorithm	GraphPad Prism	GraphPad Software	Version 10.0, RRID:SCR_002798	Statistical analysis
Software, algorithm	ImageJ	National Institutes of Health	RRID:SCR_003070	Image processing and quantification

### Reagents and chemicals

[^3^H] Gly-Sar (2 Ci/µmol) was obtained from BioScientific Pty. Ltd. (Gymea, NSW, Australia). Dulbecco’s Modified Eagle Medium (DMEM), fetal bovine serum (FBS), and other culture supplements were purchased from Thermo Scientific (Lidcombe, NSW, Australia). Gly-Sar and polymyxin B1 for molecular studies were purchased from Sapphire Biosciences (Redfern, NSW, Australia). Polymyxin B sulfate for animal studies was purchased from BetaPharm (Shanghai, China). MIPS-9541 was synthesized as previously reported (Lu et al., 2016; Deris et al., 2014; Yun et al., 2015). Plasmids containing the full-length hPepT2 and hPepT2-c-Flag were obtained from Australian Biosearch (Balcatta, WA, Australia*).* All other reagents and chemicals were purchased from Sigma-Aldrich (Castle Hill, NSW, Australia).

### Structural modeling of hPepT2 and polymyxins

The inward-open conformation of hPepT2 was predicted using AlphaFold2 via the ColabFold implementation with default parameters, employing the MMseqs2 multiple sequence alignment pipeline. To obtain the physiologically relevant outward-open conformation of hPepT2 for substrate binding, homology modeling was performed using MODELLER with the cryo-EM structure of rabbit PepT2 in the outward-open state (PDB: 7NQK) as the template. The sequence alignment between hPepT2 and rabbit PepT2 was conducted using Clustal Omega. One hundred models were generated, and the final model was selected based on the lowest Discrete Optimized Protein Energy score and verified by Ramachandran plot analysis (with >95% of residues in favored regions). The structure of polymyxin B1 was constructed and energy-minimized using the CHARMM36 force field. Computational alanine scanning of polymyxin B1 was performed by individually replacing each Dab (2,4-diaminobutyric acid) residue at positions 1, 3, 5, 8, and 9 with alanine using the mutagenesis wizard in PyMOL.

### Coarse-grained molecular dynamics (MD) simulations

Polymyxin B1 was employed as the prototype (Chan et al., 2015a) and the coarse-grained (CG) model system of hPepT2 was constructed using the Martini Maker module in CHARMM-GUI (Chan et al., 2016a). The membrane lipid composition was 50% phosphatidylcholine, 25% phosphatidylethanolamine, and 25% phosphatidylserine (Shen et al., 2022). In each simulation system, 10 coarse-grained polymyxin molecules were randomly introduced to enhance the probability of binding to hPepT2. These were solvated using CG water particles and neutralized with 0.1 M sodium chloride in a 15×15×14 nm^3^ simulation box. All CG simulations were conducted under periodic boundary conditions using the GROMACS program (version 2021.2) and the Martini 2.2 force field (Hong et al., 2010; Hong et al., 2007). To eliminate steric interference, independent steepest energy minimization was performed for every system to achieve the maximum force below 1000 kJ mol^–1^ nm^–2^. Subsequently, a six-step equilibration cycle was carried out by gradually turning off the position restraints on the lipid and polymyxin molecules. Finally, 3 µs production simulations were performed for each of two independent replicates (including independent energy minimization) were conducted for each system in the NPT (referring to the number of particles (N), system pressure (P), and temperature (T)) ensemble. Two replicates per condition were performed at this exploratory stage, as the CG-MD simulations were designed to identify the binding pathway of polymyxin B to hPepT2; the final binding poses from both replicates were highly consistent. Quantitative interaction analysis was subsequently performed using all-atom MD simulations with four replicates. The temperature and pressure were coupled to 310 K using the velocity rescaling method (time constant of 1 ps) and 1 bar using the semi-isotropic barostat and Parrinello-Rahman algorithm (time constant of 5 ps), respectively (Xu et al., 2009). Molecular visualizations, structural figures, and trajectory animations were prepared using PyMOL (Schrödinger) and ChimeraX. An animation of the CG-MD binding trajectory was provided as Video 1.

**Video 1.** An animation of the coarse-grained-molecular dynamics (CG-MD) binding trajectory of human oligopeptide transporter 2 (hPepT2) and polymyxins.

### All-atom MD simulations

The previous study of PepT_Xc_, a homolog of hPepT2, revealed that the protonation of its His67 promotes the outward-opening conformation Lin et al., 2017b; therefore, the corresponding residue His87 of hPepT2 was set to be protonated during all-atom simulations to bias the system towards an outward-opening state. In the two CG-MD replicates, polymyxin B eventually bound to the lateral opening gate of hPepT2. To examine the detailed interaction between polymyxin and hPepT2, a single polymyxin B1 molecule was docked into the predicted binding pocket of hPepT2 using AutoDock Vina (Lin et al., 2017a). Furthermore, to enhance the sampling of the binding dynamics, four different poses of polymyxin B1 were employed to build the initial hPepT2-polymyxin complex. Force field parameters for polymyxin B1 were generated using CGenFF. The membrane protein system was then constructed using the membrane builder module in CHARMM-GUI (Kumar, 2025). The membrane lipid composition was 50% phosphatidylcholine, 25% phosphatidylethanolamine, and 25% phosphatidylserine, consistent with the CG-MD simulations. Each system was solvated with TIP3P water molecules in a rectangular box of 11×11×14 nm^3^ and neutralized with 0.15 M NaCl, yielding a total of approximately 158,000 atoms.

**Figure 9.**
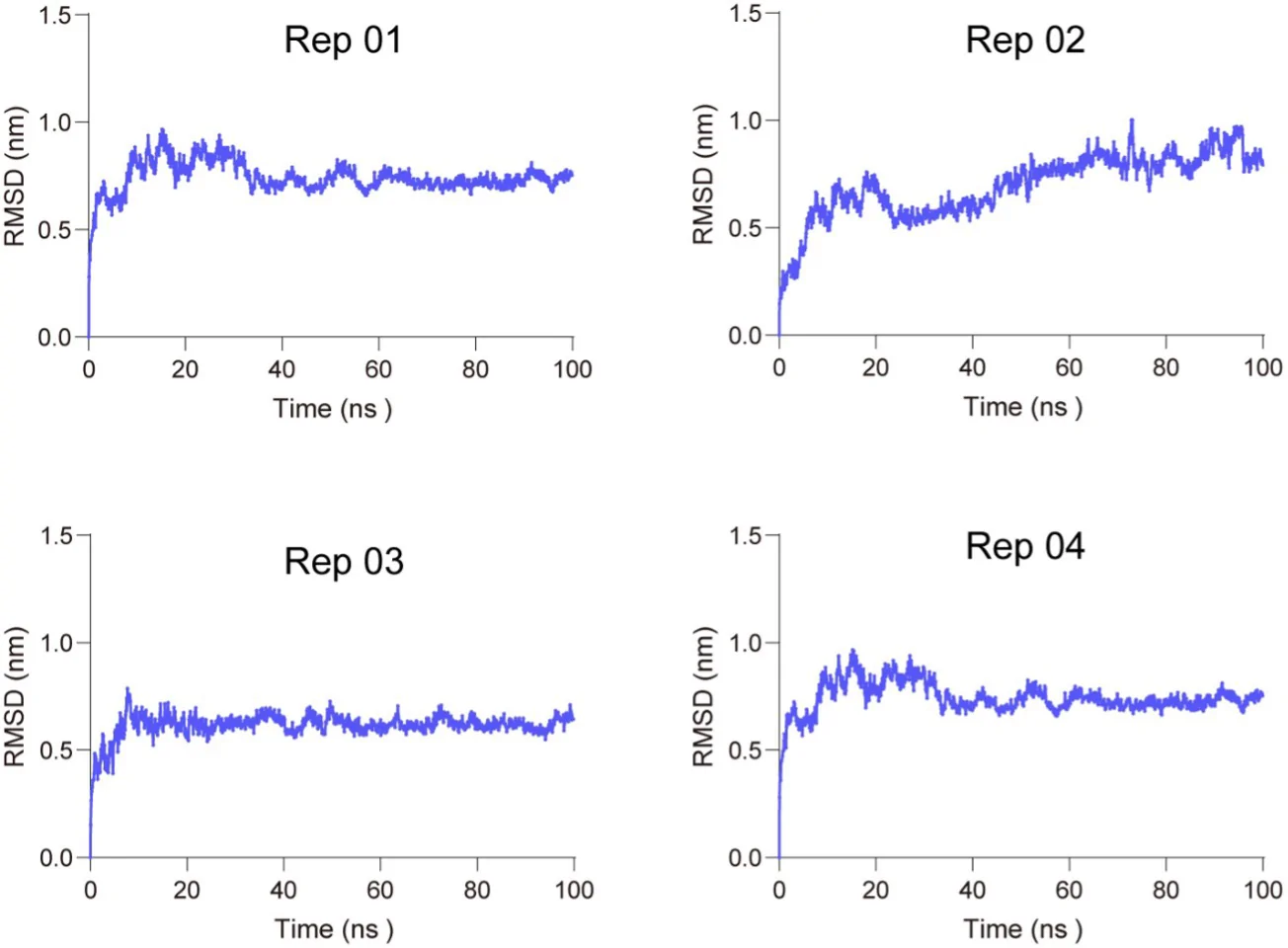
Time evolution of the root mean square deviations of polymyxin B. The results are calculated based on the four independent all-atom molecular dynamics simulation replicates.

GROMACS 2021.2 was used to perform all MD simulations with the CHARMM36 all-atom force field (Deris et al., 2012). A time step of 2 fs was employed to integrate the Newtonian equations of motion, and all bonds involving hydrogen atoms were constrained. Long-range electrostatic interactions were treated using the Particle Mesh Ewald (PME) method with a switching function applied between 10 and 12 Å (Roberts et al., 2015). Energy minimization was performed using the steepest descent algorithm. Subsequently, the system was equilibrated through a series of MD simulation steps in the NVT (referring to the number of particles (N), system volume (V), and temperature (T)) and NPT ensembles (310 K, 1 bar), during which positional restraints on membrane and protein atoms were gradually reduced. The root mean square deviations of the hPepT2-polymyxin B1 complex were calculated to assess simulation equilibration (Figure 9). No restraints were applied in the final stage of equilibration and throughout the production run. The PME method was employed to treat long-range electrostatic interactions with a short-range cut-off of 1.2 nm, while the shifted Lennard–Jones potential algorithm was used to calculate van der Waals interactions with a general cut-off of 1.2 nm and a shifting cut-off of 1.0 nm (Thombare et al., 2024; Deris et al., 2014). The trajectory during production simulations was recorded every 10 ps. Production simulations were conducted for 500 ns for outward-open conformation sampling and 100 ns for polymyxin-hPepT2 interaction analysis.

### Site-directed mutagenesis and plasmid DNA sequencing

The hPepT2 mutants were generated using a QuickChange II site-directed mutagenesis kit (Agilent Technologies, Mulgrave, Victoria, Australia) with the primers listed in Table 4. All mutant construct sequences were confirmed by DNA sequencing (Ramaciotti Centre for Gene Function Analysis, Randwick, NSW, Australia) with the BigDye Terminator v3.1 kit (Thermo Scientific, Lidcombe, NSW, Australia) (Chan et al., 2016b; Chan et al., 2015a; Zheng et al., 2014; Zhou et al., 2013; Zhou et al., 2010).

### Transfection of hPepT2 and its mutants into HEK293 cells

HEK293T cells were cultured in DMEM supplemented with 10% FBS at 37 °C with 5% CO_2_. HEK293T cell line was authenticated by the Australian Genome Research Facility (Urrbrae, SA, Australia) and checked for mycoplasma every 3 months. Cells were transfected with hPepT2 or its mutant plasmid DNA using Lipofectamine 2000 Reagent according to the manufacturer’s instructions (Invitrogen, Mount Waverley, VIC, Australia) (Lu et al., 2018; Lu et al., 2016; Chan et al., 2016a; Ali et al., 2021; Li et al., 2014). At 24 hr after transfection, the cellular uptake of [^3^H] Gly-Sar or MIPS-9541 was assessed.

### Transport uptake assay

To evaluate the uptake of [^3^H] Gly-Sar via hPepT2 or its mutant transporters, we measured the accumulation of [^3^H] Gly-Sar in hPepT2-overexpressing HEK293 cells. The uptake was conducted at 37 °C in phosphate-buffered saline (PBS, pH 5.0) containing 5 mM glucose. The specific substrate concentration was 2.5 μM [^3^H] Gly-Sar, and the duration was 8 min according to our previous studies (Lu et al., 2018; Lu et al., 2016). The uptake assay was terminated by three rapid washes with ice-cold PBS. Samples were lysed in 0.2 M NaOH, neutralized in 0.2 M HCl, and then processed for liquid scintillation counting. The uptake counts for hPepT2 or its mutant-expressing cells were all subtracted by those of the control.

**Figure 10.**
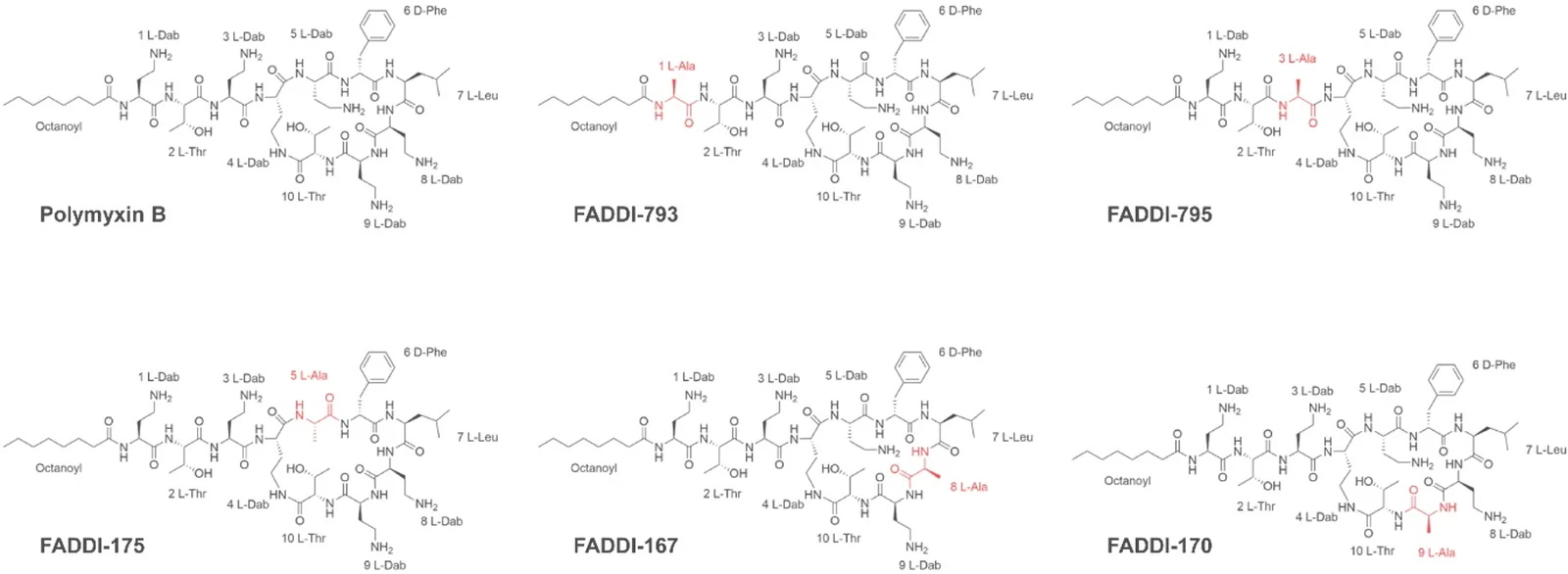
Chemical structures of polymyxin B and its analogs. The modified structural moieties are labeled in red.

To evaluate the uptake of polymyxin B via hPepT2 or its mutant transporters, we measured the accumulation of a validated fluorescent polymyxin probe MIPS-9541 and polymyxin analogs (i.e. FADDI-167,–170, −175,–793, and –795, Figure 10) in hPepT2-overexpressing HEK293 cells (Lu et al., 2016). The uptake was performed in PBS (pH 5.0) supplemented with 5 mM glucose for 10 min at 37 °C. Intracellular fluorescence accumulation was quantified with excitation/emission wavelengths of 350 nm/518 nm using a Tecan Safire II microplate reader (Thermo Scientific, Lidcombe, NSW, Australia). Background counts of the vector-transfected cells were subtracted from all uptake measurements. Kinetic analysis was conducted by measuring the uptake of transporter-expressing cells with a range of concentrations of MIPS-9541 (0–50 µM) over 10 min. Apparent K_m_ and V_max_ values were determined using GraphPad Prism 10.0 (Lu et al., 2016; Chan et al., 2015b).

### Fluorescence imaging of MIPS-9541

HEK293 cells were seeded on chamber slides and transfected with hPepT2 or its mutant plasmids. After 24 hr, the culture medium was removed, and cells were incubated with freshly prepared MIPS-9541 solution in PBS (10 µM, pH 5.0) for 10 min at 37 °C. Cells were then washed with ice-cold PBS three times and mounted in SlowFade Gold Antifade Mountant reagent supplemented with 4',6-diamidino-2-phenylindole (DAPI, Thermo Scientific, Lidcombe, NSW, Australia). Samples were imaged with a Leica Thunder 3D Imager (Leica Microsystems, North Ryde, NSW, Australia).

### Cell surface biotinylation

We employed sulfo-NHS-SS-Biotin (Campbell Science, Rockford, IL, USA) to label the plasma membrane proteins in HEK293 cells overexpressing hPepT2 or its mutants (Zhou et al., 2007). Cell culture plates were pre-chilled on ice. Culture medium was aspirated, and cells were washed twice with cold PBS (pH 8.0). Freshly prepared 1 mg/mL sulfo-NHS-SS-Biotin in PBS (pH 8.0) was then added to each well and incubated for 30 min. Cells were washed twice with 100 mM glycine in PBS (pH 8.0) and then three times with PBS (pH 7.4). Cells were lysed in the lysis buffer (Tris 10 mM, NaCl 150 mM, EDTA 1 mM, SDS 0.1%, and Triton X-100 1% with 1:1000 dilution of a protease inhibitor cocktail) (Chan et al., 2015a). Cell lysate was subjected to centrifugation at 14,000 *g* and 4 °C for 10 min, and the supernatant was collected. Pre-washed Streptavidin-Agarose beads were supplied to the samples and incubated with rotation at 4 °C for 1 hr. After three rounds of centrifugation and washing with PBS (pH 7.4), beads were resuspended in PBS with 1 × Laemmli buffer containing 2.5% β-mercaptoethanol and incubated at 55 °C for 30 min. The remaining supernatant was collected, denatured, and subjected to electrophoresis.

### Total cell lysis, electrophoresis, and immunoblotting

Upon washing, cells were collected and lysed in the lysis buffer. After incubating on ice for 10 min, cell lysate was centrifuged at 14,000 *g* and 4 °C for 10 min. The supernatant was collected as total cell lysate and denatured at 55 °C in 1 × Laemmli buffer containing 2.5% β-mercaptoethanol for 30 min.

Protein lysate samples were loaded onto 10% SDS-PAGE gels followed by electrophoresis as described before (Zhou et al., 2008), and the protein samples on the gels were transferred to polyvinylidene difluoride (PVDF) membranes. Following blocking with 5% non-fat milk in PBS-Tween (Na_2_HPO_4_ 80 mM, KH_2_PO_4_ 20 mM, NaCl 100 mM, and 0.05% Tween 20, pH 7.5) and multiple washings with PBS-Tween, the immunoblots were incubated at 4 °C overnight with anti-flag (DYKDDDDK) antibody (1:1000; Catalog Number 2368, Genesearch, Arundel, QLD, Australia) or anti-β-actin antibody (1:2000, Catalog Number 4967, Genesearch). After washing with PBS-Tween three times, the immunoblots were incubated at room temperature for 1 hr with goat anti-rabbit IgG conjugated with HRP (1:5000; Catalog Number A0545, Sigma-Aldrich) or goat anti-mouse IgG conjugated with HRP (1:10,000; Catalog Number A2554, Sigma-Aldrich). The blots were washed, incubated with Immobilon Western Chemiluminescent HRP substrate (Merck Millipore, Kilsyth, VIC, Australia), and imaged with an ImageQuant LAS 500 (GE Healthcare, Avantor, PA, USA). The densitometry analysis of immunoblots was processed with ImageJ (NIH, Bethesda, MD, USA).

### Chemical synthesis of polymyxin analogs

Five lipopeptides (FADDI-167, FADDI-170, FADDI-175, FADDI-793, and FADDI-795) were synthesized and purified in-house as described before (Roberts et al., 2022; Jiang et al., 2021). Briefly, the lipopeptides were prepared using a Protein Technologies Prelude automated peptide synthesizer according to the standard protocol for Fmoc solid-phase peptide chemistry.

### Measurements of minimum inhibitory concentrations

MICs were measured using the broth micro-dilution method with clinical isolates and American Type Culture Collection (ATCC) strains (Roberts et al., 2015). Each bacterial strain (~10^6^ colony-forming units [CFU] per mL) was suspended in 100 μL of freshly prepared cation-adjusted Mueller-Hinton broth (CaMHB) in 96-well plates. Lipopeptides were diluted to different concentrations in CaMHB and added to the plates for subsequent incubation at 37 °C for 18–24 hr. MICs were determined as the lowest concentration at which the visible growth of bacteria was inhibited.

### Nephrotoxicity in mice

The ARRIVE guideline was followed for in vivo experiment reporting. The mouse nephrotoxicity study was approved by the Monash Animal Ethics Committee. Mice (Swiss, female, 6–8 weeks old) were maintained in micro-isolators in a temperature-controlled PC2 animal laboratory with an ambient humidity of 50–70% and a 12 hr/12 hr dark/light cycle. Mice were randomly divided into groups. Lipopeptide solutions were subcutaneously administered to mice with a dose of 8 mg/kg every 2 hr for 6 doses in one day (a total dose of 48 mg/kg/day, n=3 each group). At 24 hr after the last injection, mice were sacrificed, the kidneys were collected from mice immediately, and fixed in 10% formalin buffer (pH 7.4, Sigma-Aldrich, Australia). Histological examination of the kidney samples was conducted at the Australian Phenomics Network-Histopathology and Organ Pathology (University of Melbourne, Parkville, VIC, Australia). An experienced pathologist blinded to treatment groups assessed renal histology and graded the severity of nephrotoxicity using a semi-quantitative scoring system (scores 1–10) as previously described (Roberts et al., 2022). The percentage of kidney affected was also scaled in the range of 0–6 (0:<1%; 1: 1 to <5%; 2: 5 to <10%; 3: 10 to <20%; 4: 20 to <30%; 5: 30 to ≤40% and 6:>40%). A semi-quantitative score (SQS) was calculated using the grade score and kidney damage percentage to quantify the overall kidney damage (Roberts et al., 2022). Noteworthy, in this proof-of-concept study, we employed acute kidney damage analysis in a small number of mice to rapidly screen these analogs for nephrotoxicity. Three biological replicates were used because our mouse nephrotoxicity model is robust, as demonstrated in our drug discovery program (Roberts et al., 2022). Moreover, minimizing animal use is a fundamental principle of the 3Rs (Replacement, Reduction, and Refinement).

### Statistical analyses

Unpaired *t*-test was employed to detect the variance between the protein expression of hPepT2 and its mutants. Welch’s *t*-test was used to assess the differences between the transport function or kinetic parameters of hPepT2 and its mutants. All data are presented as mean± standard deviation (SD), and a p-value <0.05 was considered statistically significant. All experiments were repeated three times with triplicates in each experiment.

## Data Availability

All strains involved in this study are available upon request. All data analyzed in the current study are provided in the manuscript and supporting files.

## Additional files

MDAR checklist
