# Supplementary material for: Lateral opening site of human oligopeptide transporter 2 plays a key role in the interaction with polymyxins
Source: eLife. 2026 Oct 29;15:RP111387. doi: 10.7554/eLife.111387 (PMC13719170; doi:10.7554/eLife.111387)
Supplement: Supplementary file 1 [file elife-111387-fig6-data1.pdf]

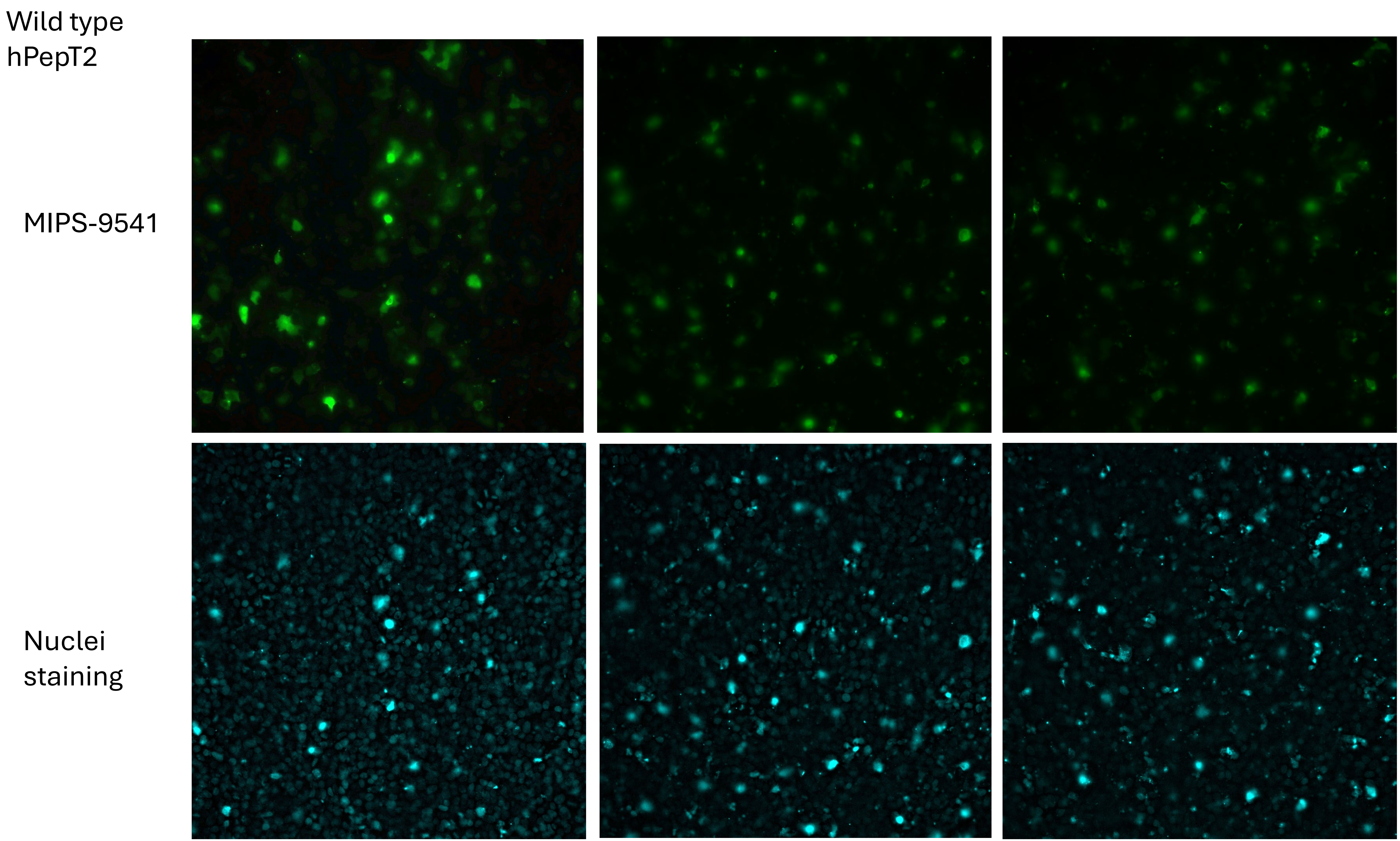
Wild type
hPepT2
MIPS-9541
Nuclei
staining

hPepT2
214A

MIPS-9541

Nuclei
staining

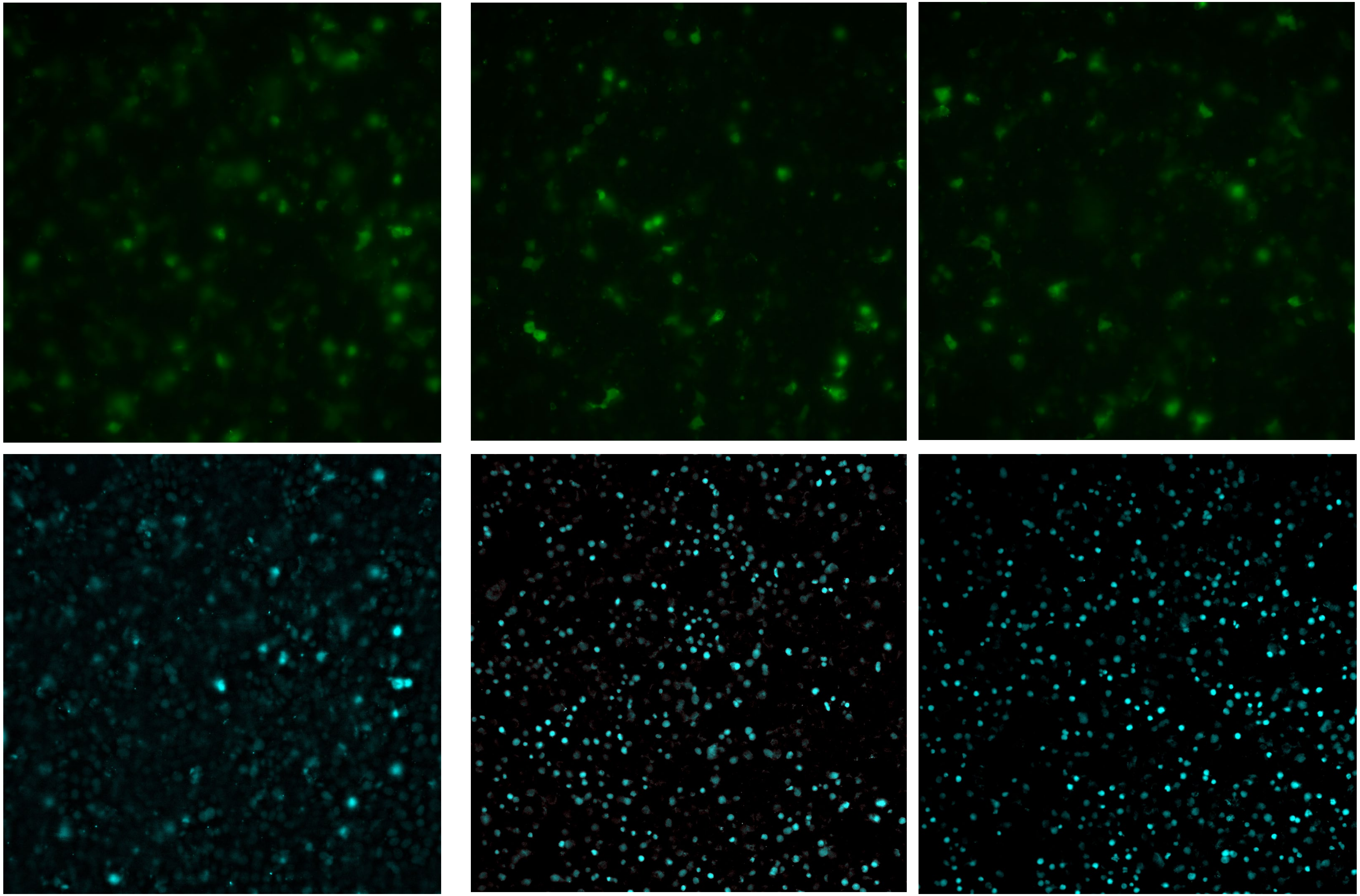

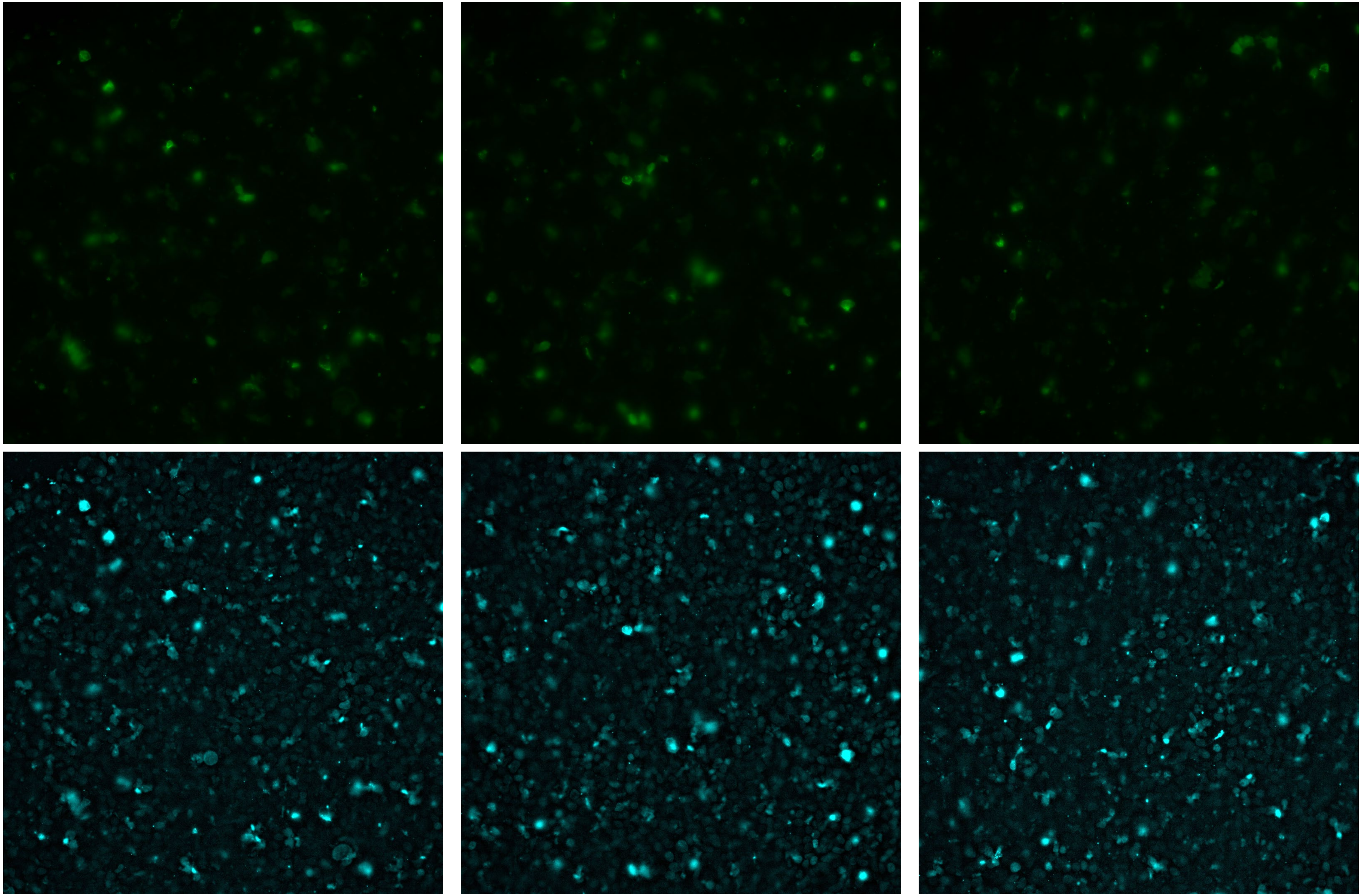
hPepT2
215A
MIPS-9541
Nuclei
staining

hPepT2
342A

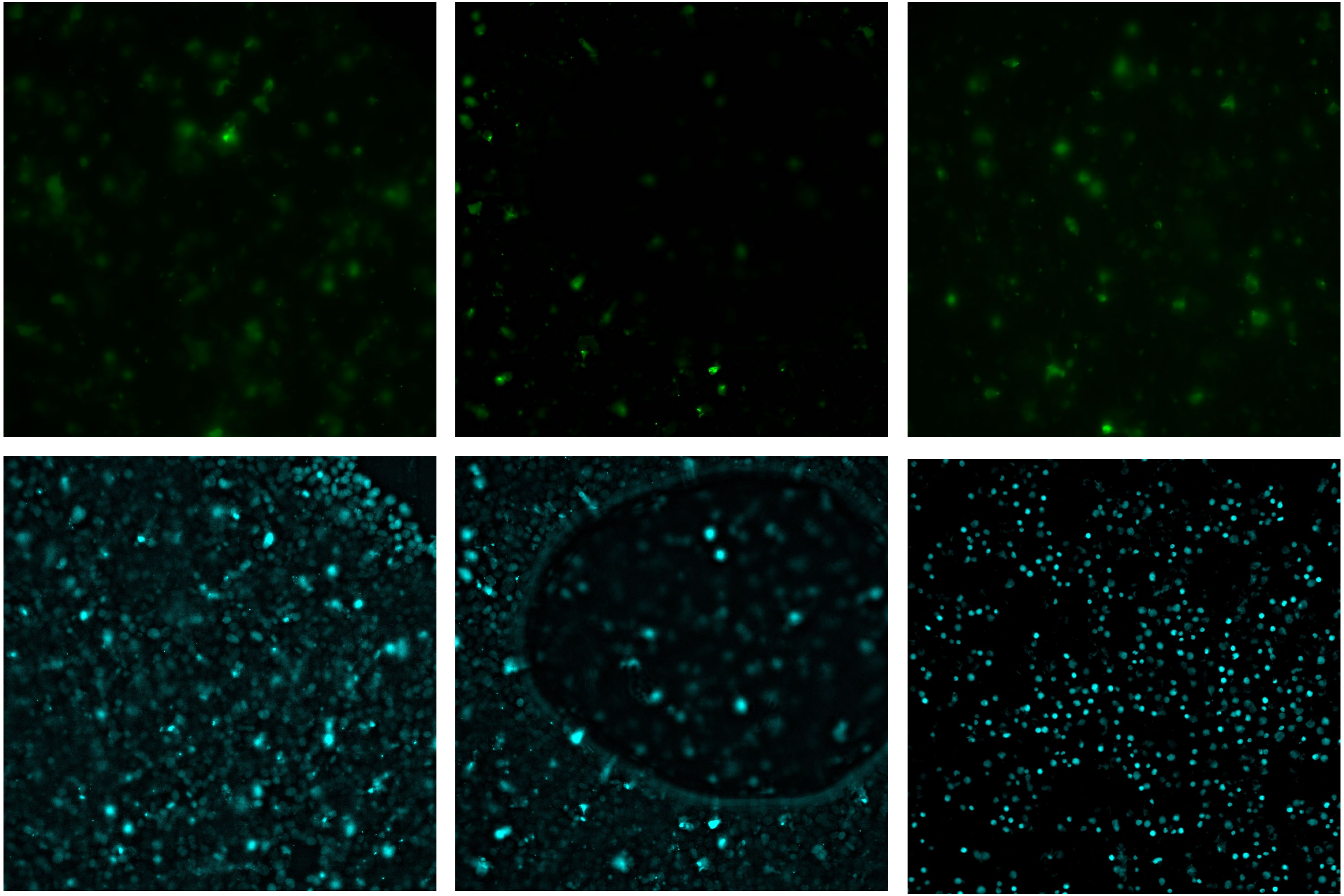

MIPS-9541

Nuclei
staining

hPepT2
555A

MIPS-9541

Nuclei staining

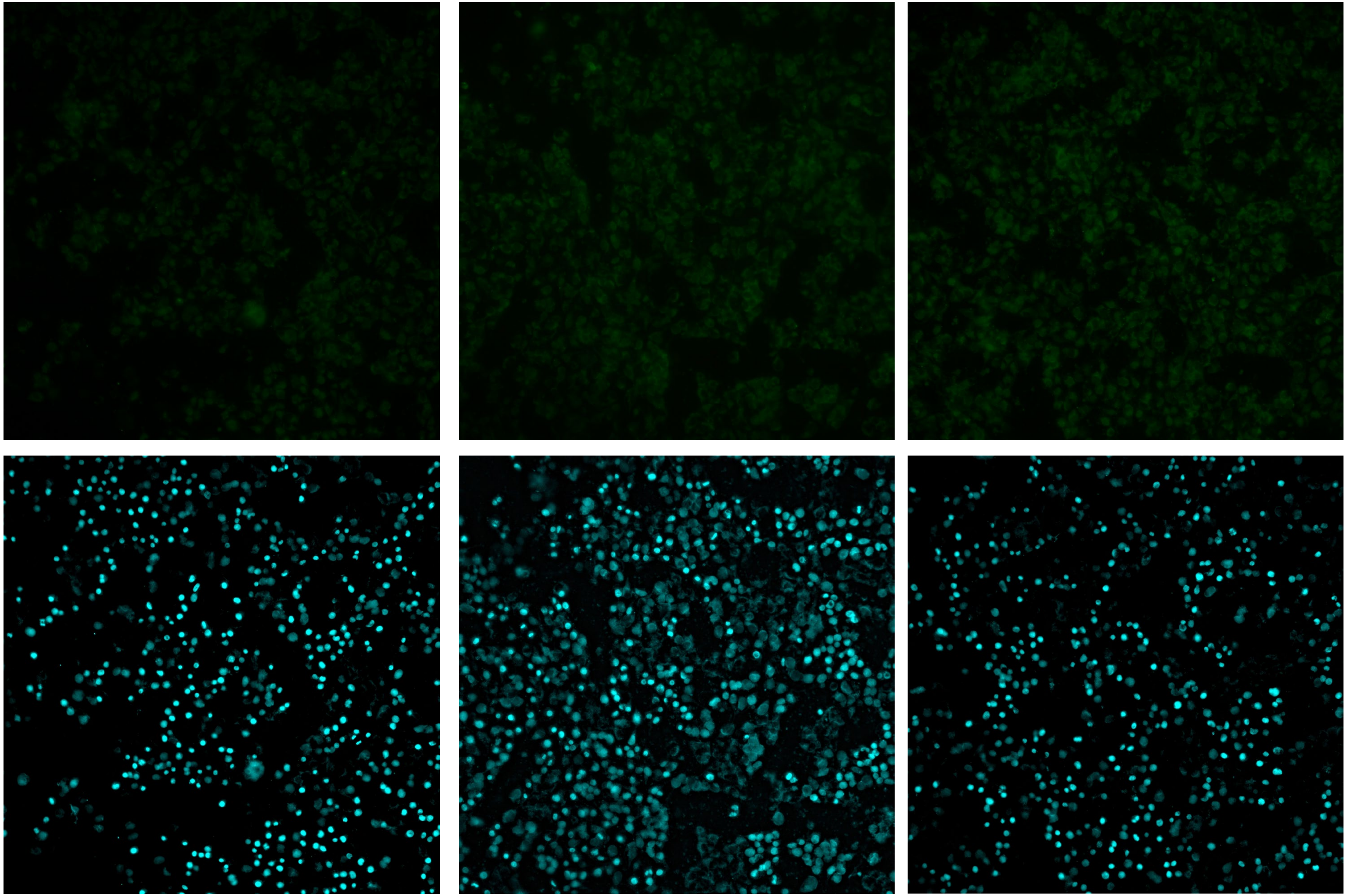

hPepT2
622A

MIPS-9541

Nuclei staining

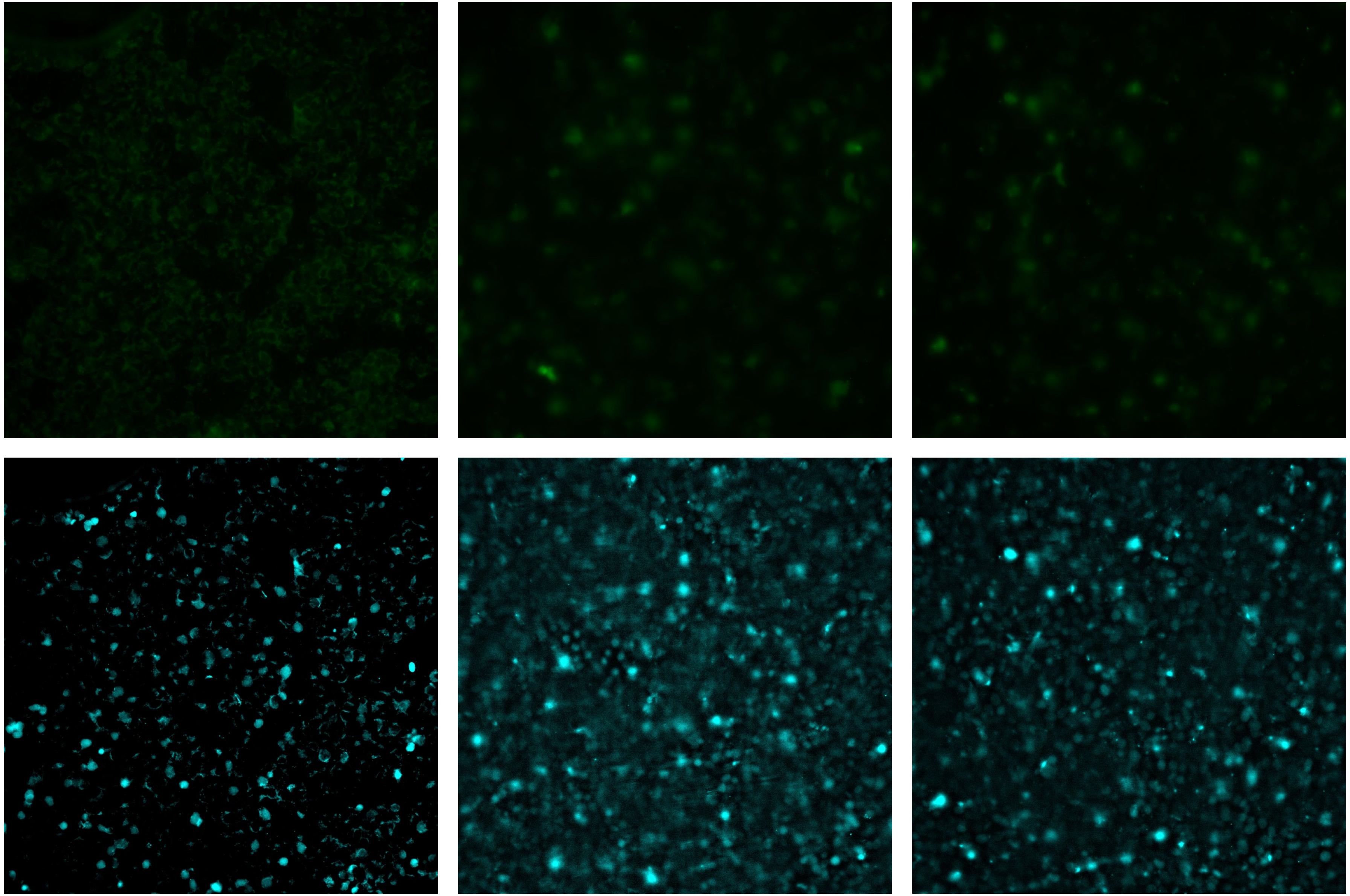

NC

MIPS-9541

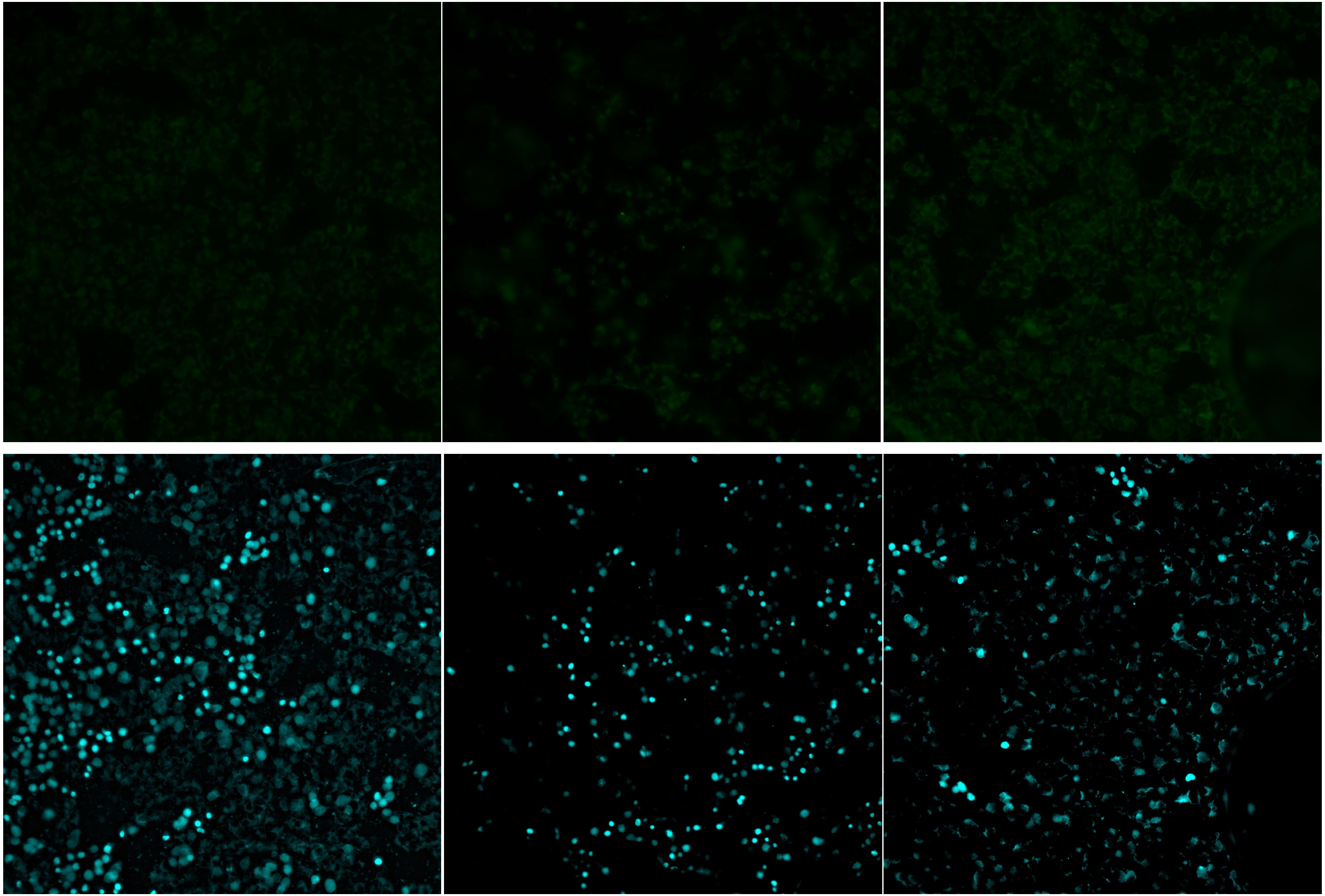

Nuclei staining

Fig. 6 source data 1. The original fluorescence images taken for hPepT2, its mutants and vector expressing HEK293 cells

The upper panels are images taken for MIPS-9541; the bottom panels are images taken for nuclei staining. NC: vector transfected negative control.
