# Supplementary material for: Lateral opening site of human oligopeptide transporter 2 plays a key role in the interaction with polymyxins
Source: eLife. 2026 Oct 29;15:RP111387. doi: 10.7554/eLife.111387 (PMC13719170; doi:10.7554/eLife.111387)
Supplement: Supplementary file 2 [file elife-111387-table2-data1.pdf]

**Chemical information**					
	**Polymyxin B**	**FADDI-170#**	**FADDI-175#**	**FADDI-793#**	**FADDI-795#**
**Sequence**	Octanoyl-Dab-Thr-Dab-Dab*-Dab-D-Phe-Leu-Dab-Ala-Thr*	Octanoyl-Dab-Thr-Dab-Dab*-Dab-D-Phe-Leu-Dab-Ala-Thr*	Octanoyl-Dab-Thr-Dab-Dab*-Ala-D-Phe-Leu-Dab-Dab-Thr*	Octanoyl-Ala-Thr-Dab-Dab*-Dab-D-Phe-Leu-Dab-Dab-Thr*	Octanoyl-Dab-Thr-Ala-Dab*-Dab-D-Phe-Leu-Dab-Dab-Thr*
**Amount**	15.0 mg (94.8%)	15.0 mg (94.8%)	21.0 mg (89.8%)	60.5 mg (97.9%)	68.2 mg (98.7%)
**MW (free base)**	1160.4	1160.4	1160.4	1160.4	1160.4
**Salt form**	(TFA) 1616.4	(TFA) 1616.4	(TFA) 1616.4	(TFA) 1616.8	(TFA) 1616.8
**Structural Modifications**	L-Ala position 9	L-Ala position 9	L-Ala position 5	L-Ala pos 1	L-Ala pos 3
***In vitro* efficacy**	**MIC (μg/mL)**				
**Polymyxin-susceptible bacterial isolates**					
P. aeruginosa					
ATCC 27853	1	2	>32	2	4
FADDI-PA022	1	4	>32	4	8
FADDI-PA025	2	32	>32	8	32
A. baumannii					
ATCC 19606	1	>32	>32	>32	8
FADDI-AB034	0.25	>32	>32	>32	4
ATCC 17978	0.5	32	>32	16	4
K. pneumoniae					
ATCC 13883	16	>32	>32	4	4
FADDI-KP032	0.5	4	>32	4	1
E. cloacae					
FADDI-EC006	0.5	8	>32	8	0.5
FADDI-EC001	0.5	8	32	2	1
FADDI-EC003	<0.125	8	32	4	0.5
***In vivo* efficacy**					
**Nephrotoxicity**					
Dose regimen	IP, 8mg/kg, a dose/2h, 6 doses in one day	IP, 8mg/kg, a dose/2h, 6 doses in one day	NA	IP, 8mg/kg, a dose/2h, 6 doses in one day	IP, 8mg/kg, a dose/2h, 6 doses in one day
Total dose (mg/kg)	48	48	NA	48	48
Kidney Histology Damage Score (overall score)			NA		
Mouse 1	4.0	2.0	NA	0.2	0.1
Mouse 2	5.0	0.2	NA	0.2	0.2
Mouse 3	6.0	0.2	NA	4.0	0.0
Max Kidney SQS obs	+1	+1	NA	+1	0
Treatment period (day	1.5	1.5	NA	1.5	1.5
Number of mice testec	3	3	NA	3	3
**Acute toxicity**					
IV (Bolus) MTD (mg/kg)	5.0	5.7	0.5	7.2	5.0
No. of mice	1	1	1	1	1

Reference

#Jiang, Xukai, et al. "A novel chemical biology and com approach to expedite the discovery of new-generation against life-threatening*Acinetobacter baumannii*." *Che Science*, vol. 12, no. 36, 2021, pp. 12211-12220, https://doi.org/10.1039/D1SC03460J
