# Supplementary material for: Lateral opening site of human oligopeptide transporter 2 plays a key role in the interaction with polymyxins
Source: eLife. 2026 Oct 29;15:RP111387. doi: 10.7554/eLife.111387 (PMC13719170; doi:10.7554/eLife.111387)
Supplement: Supplementary file 5 [file elife-111387-fig7-data2.zip › figure 7 source data 2/Figure 7 source data 2.pdf]

Figure 7a

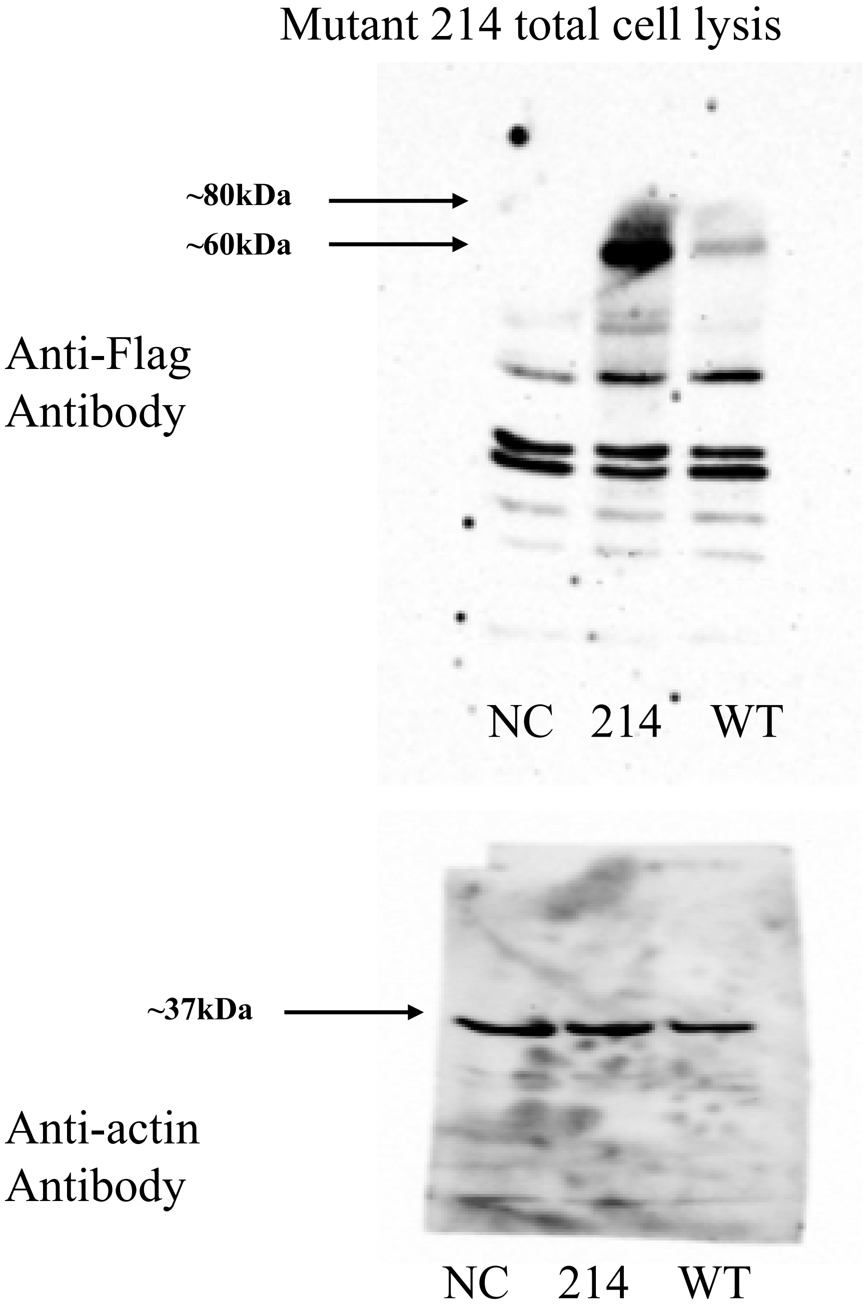

Figure 7b

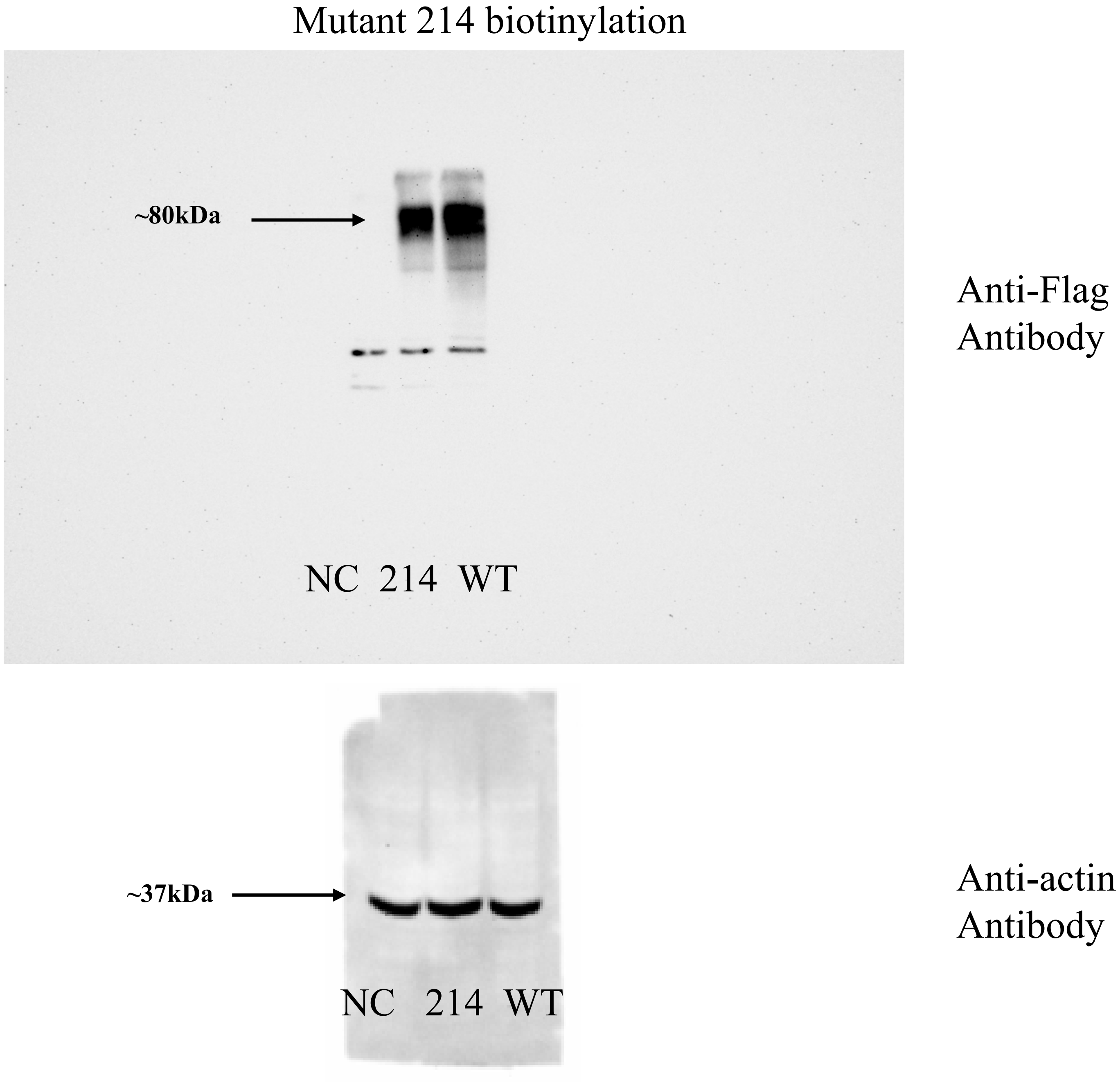

Figure 7a

Mutant 215 total cell lysis

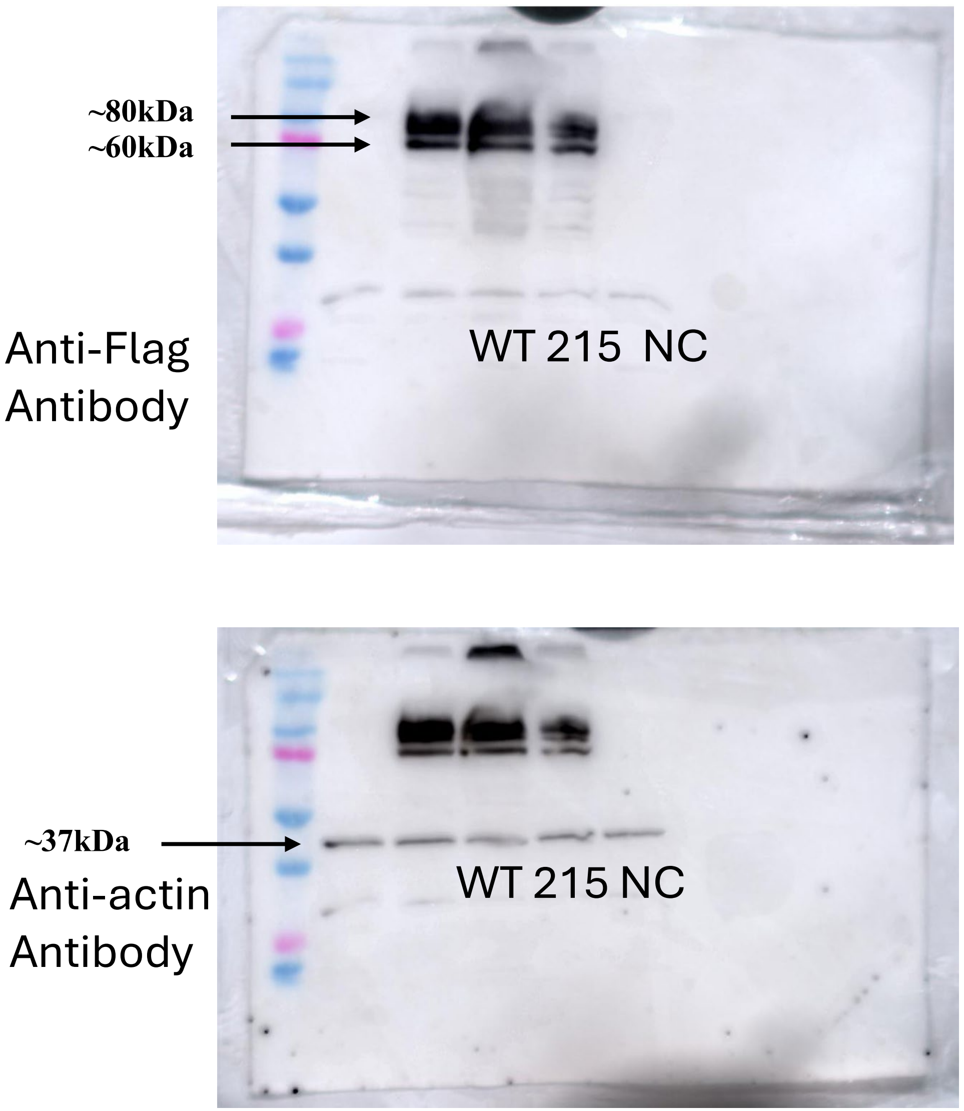

Figure 7b

Mutant 215 biotinylation

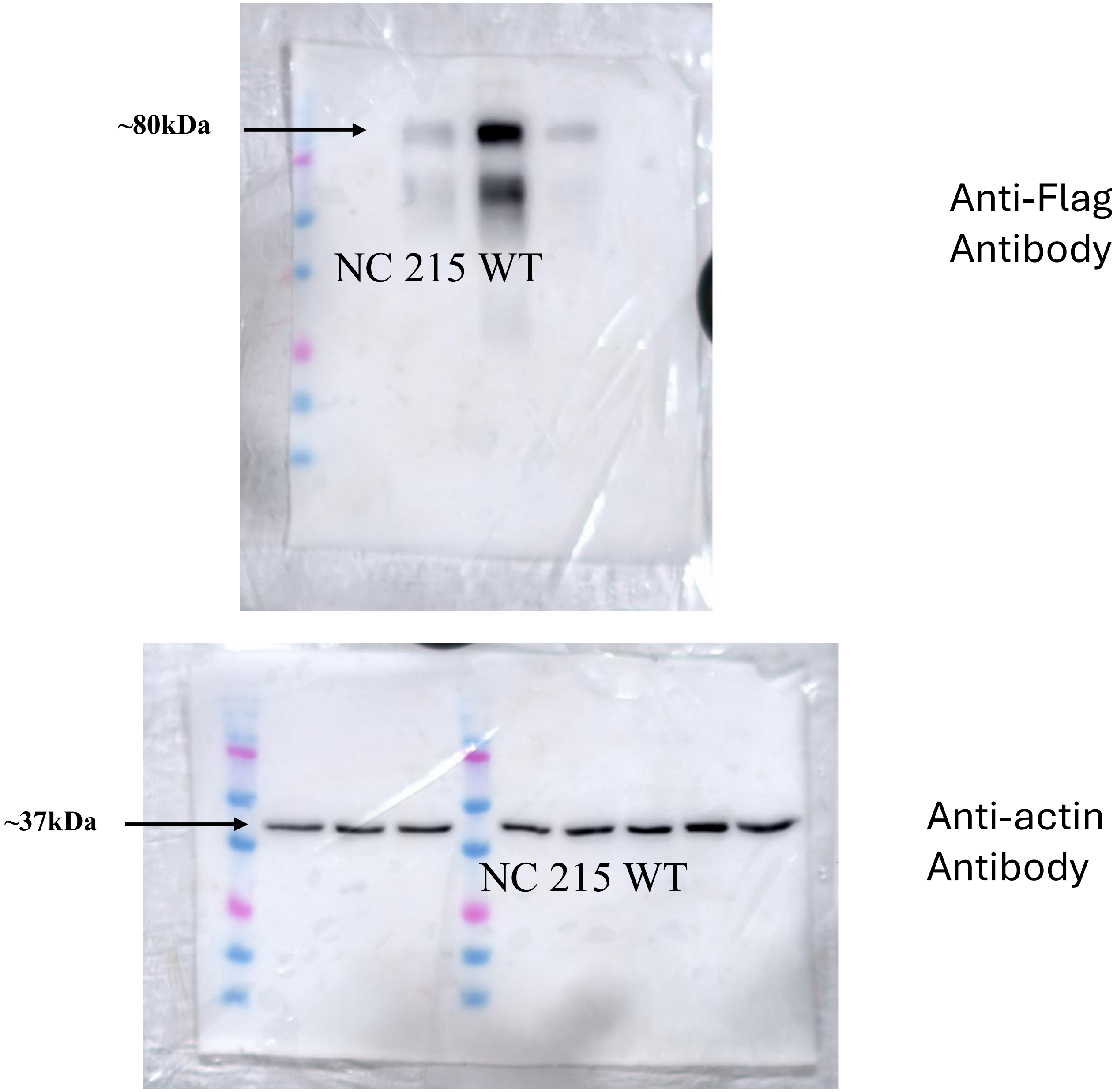

Figure 7a

Mutant 342 total cell lysis

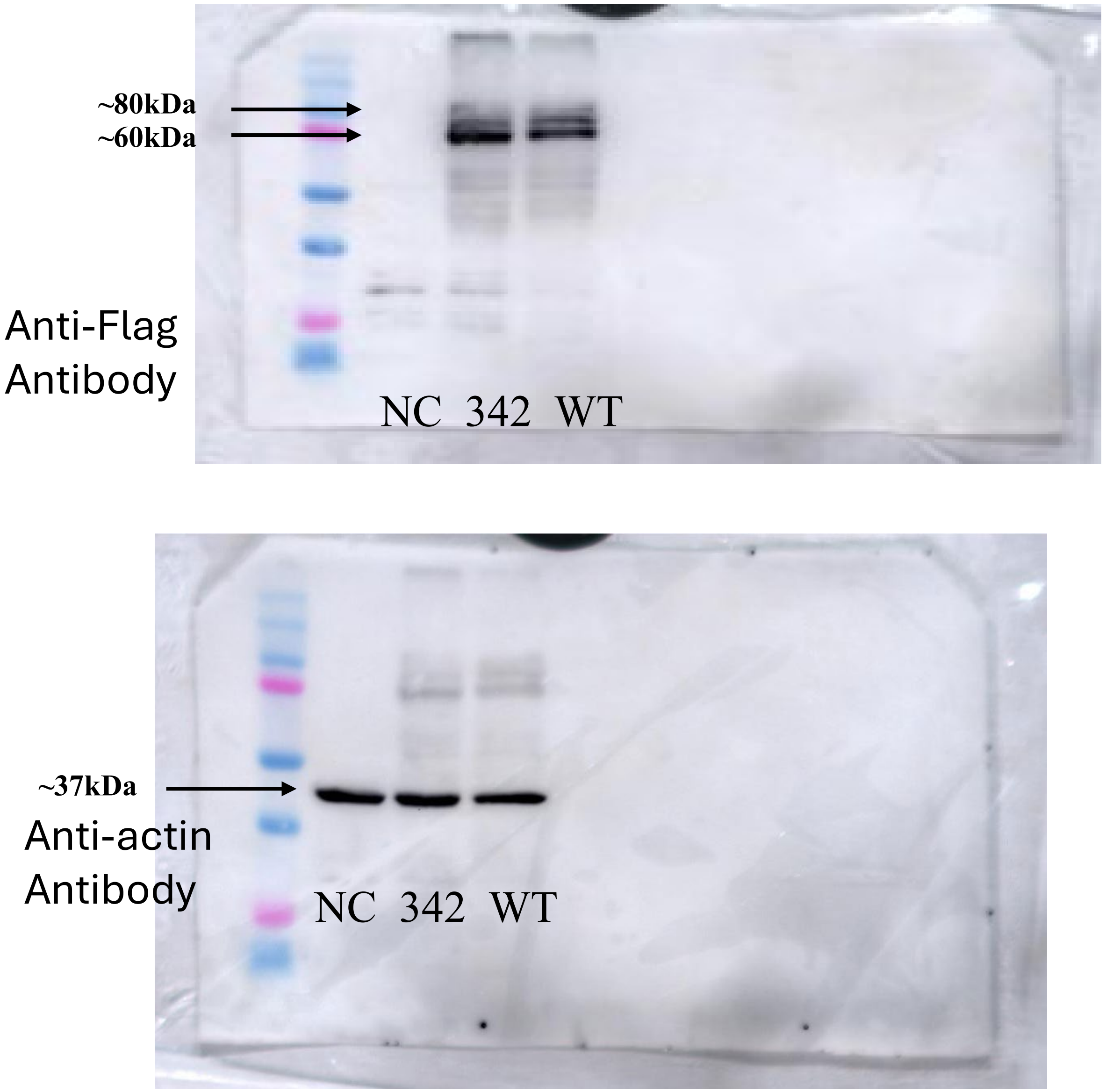

Figure 7b

Mutant 342 biotinylation

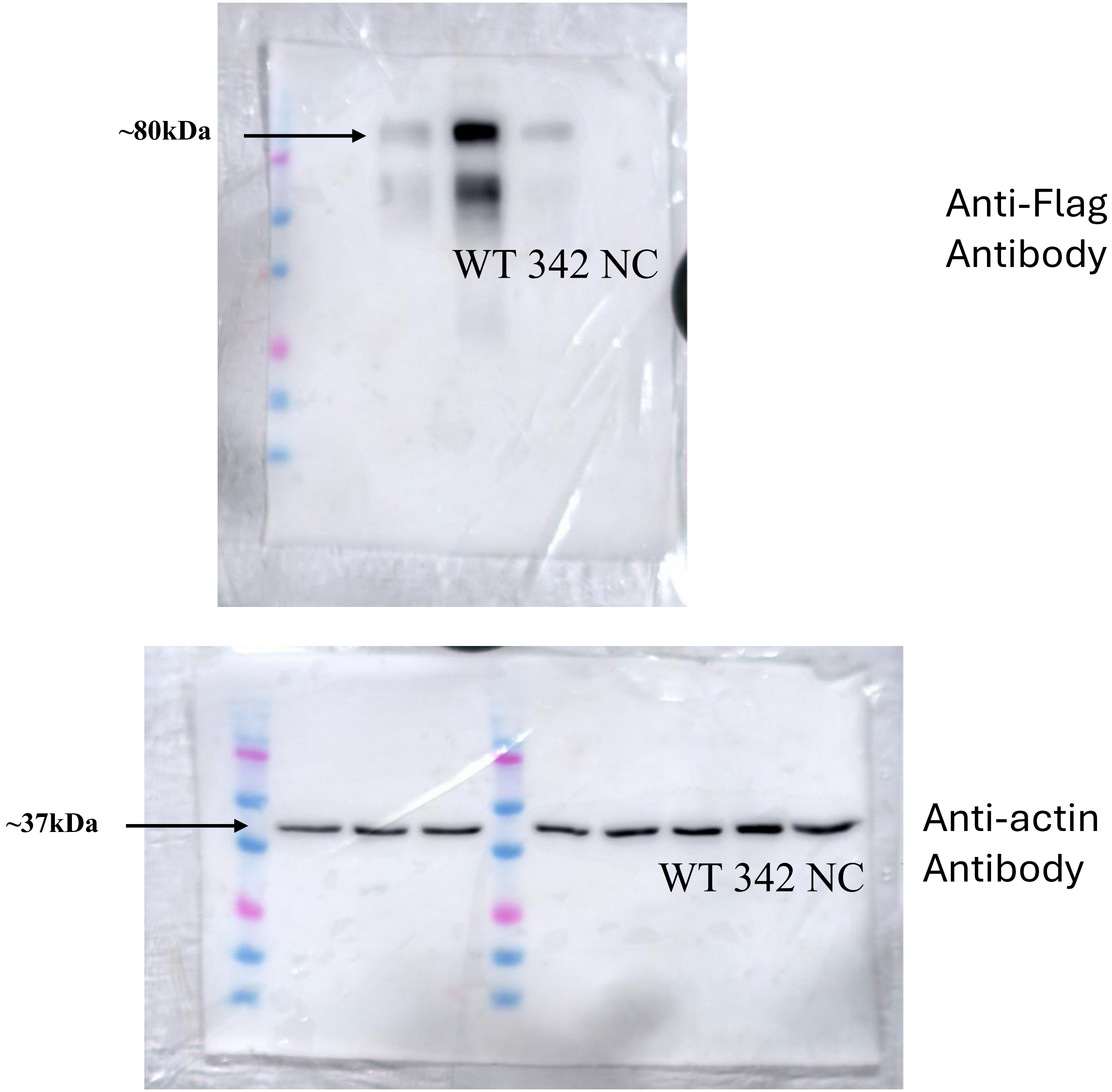

Figure 7a

Mutant 555 total cell lysis

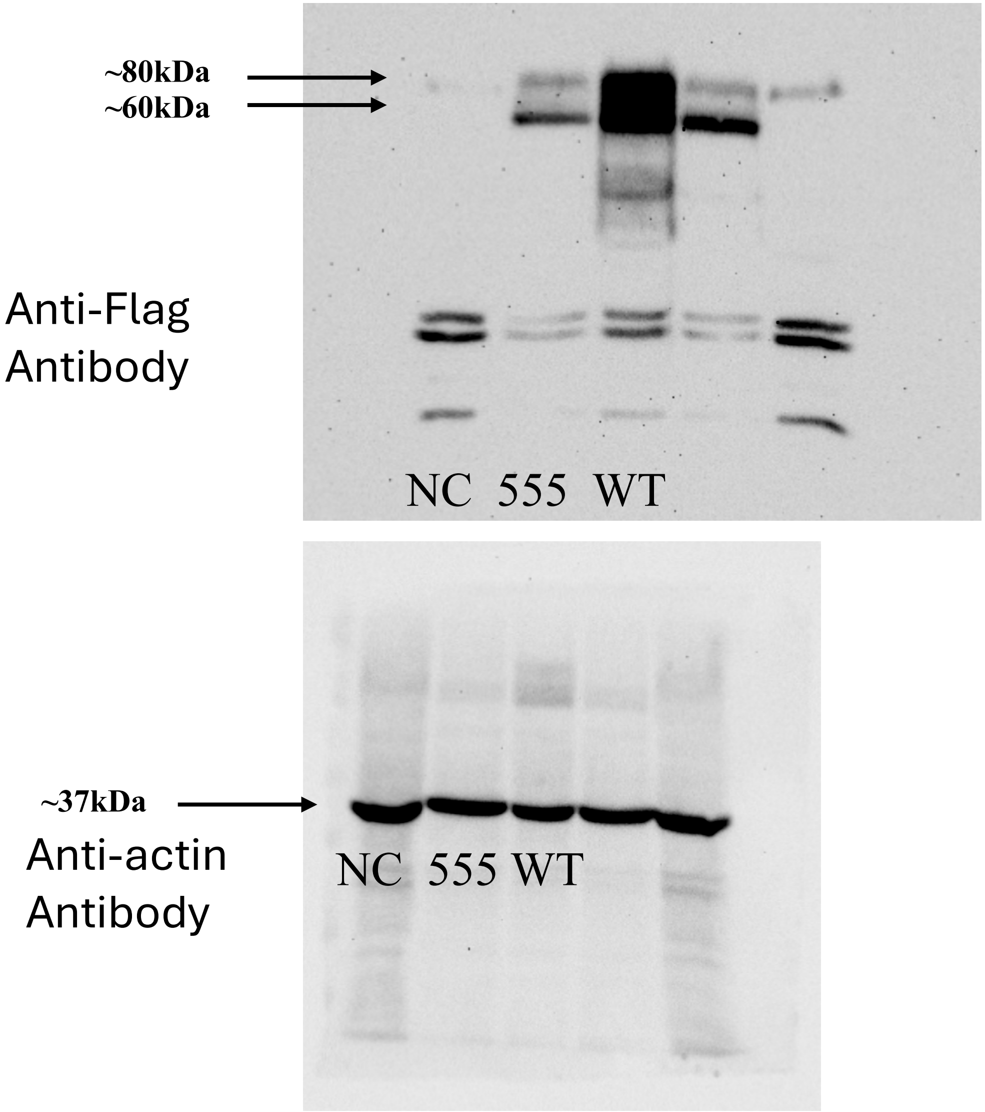

Figure 7b

Mutant 555 biotinylation

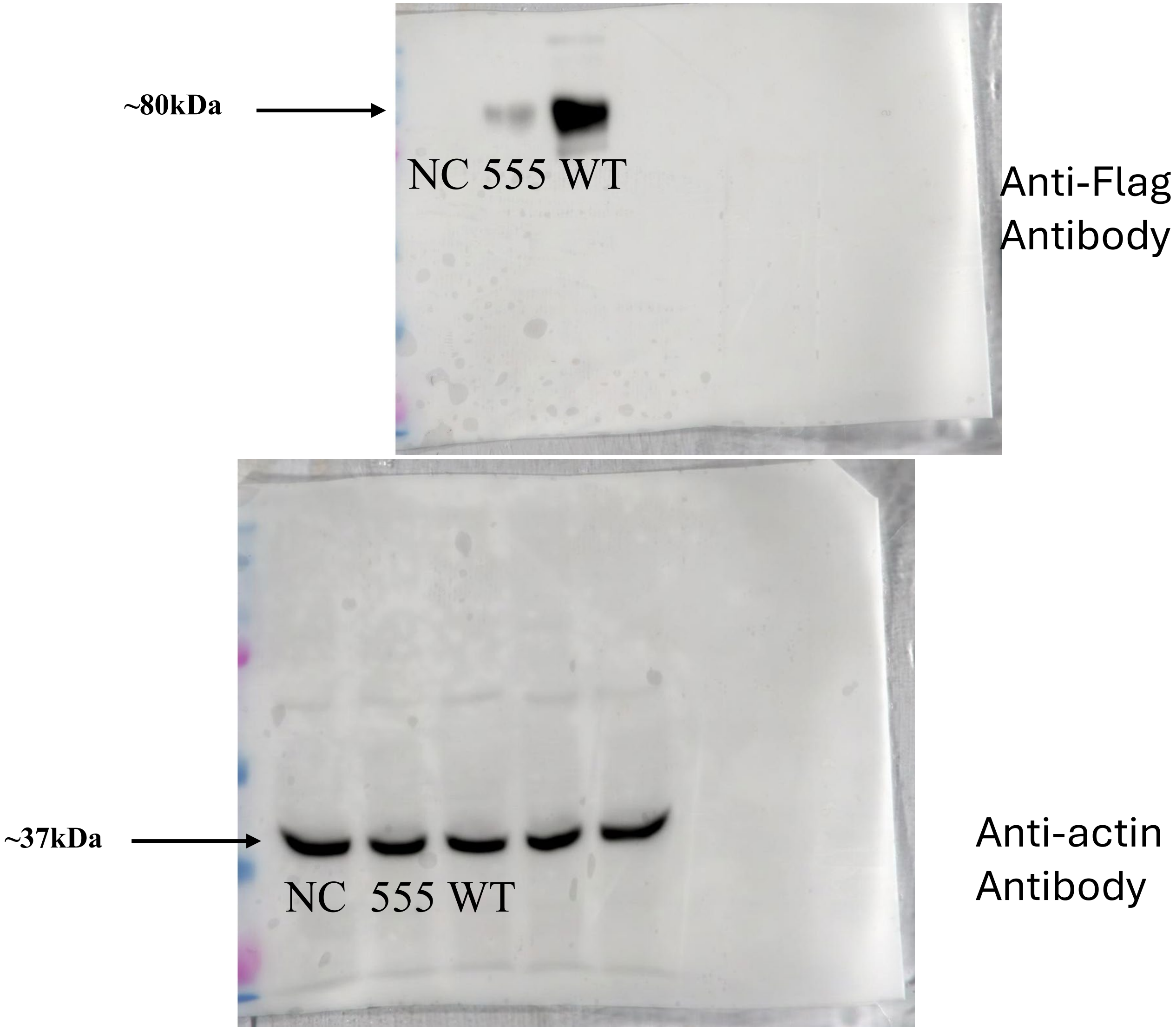

Figure 7a

Mutant 622 total cell lysis

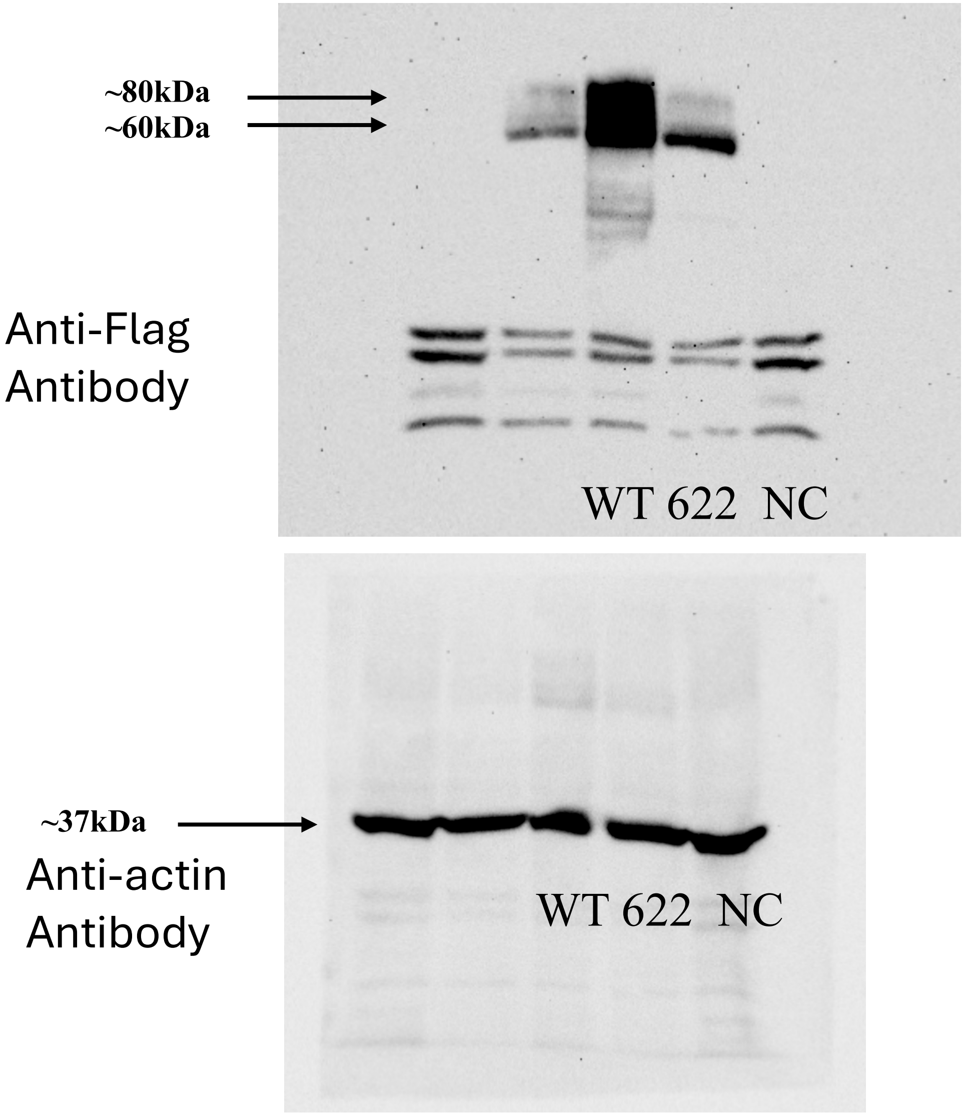

Figure 7b

Mutant 622 biotinylation

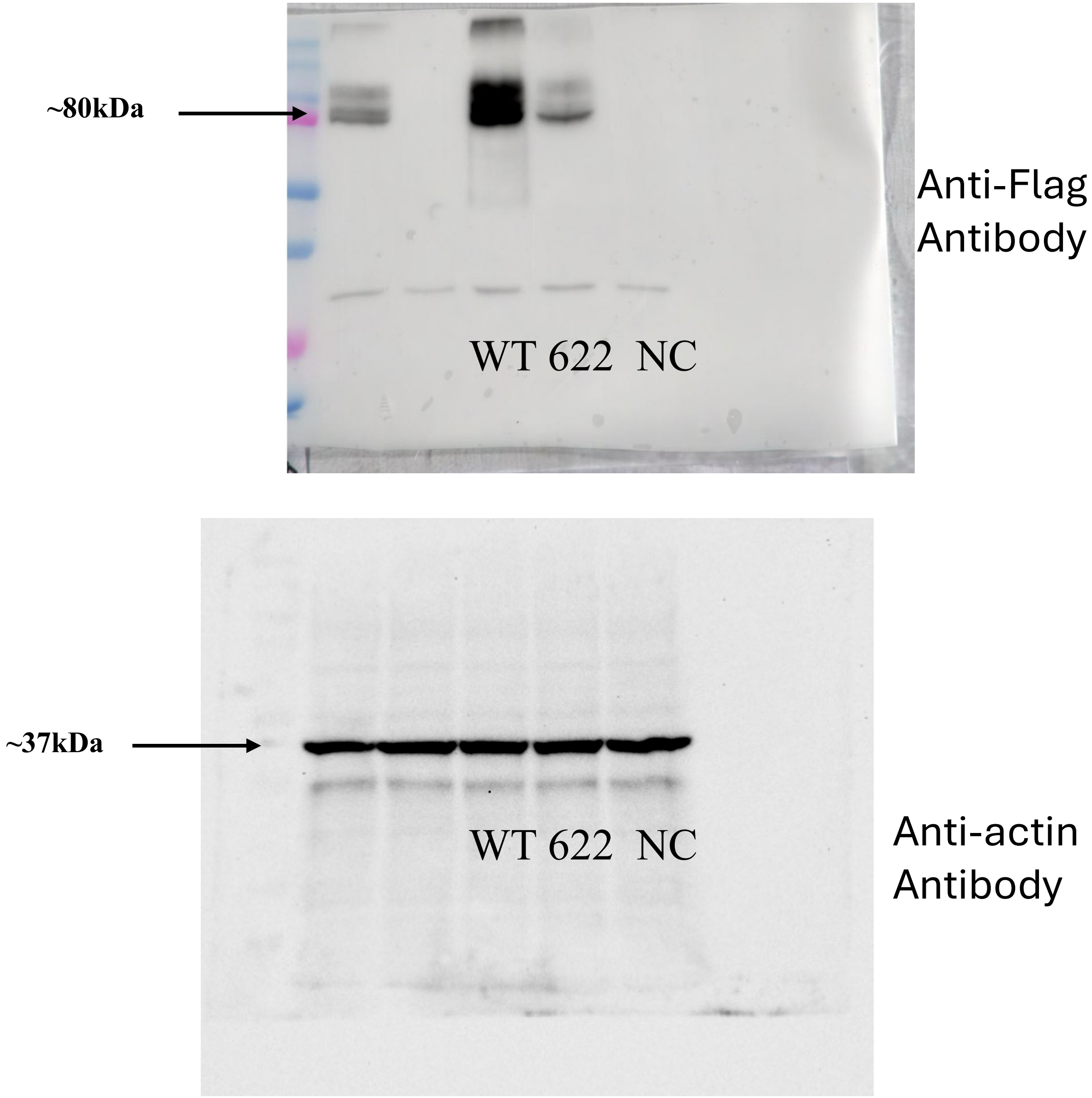

Figure 7, Source Data 2. Figures with the uncropped gels or blots with the relevant bands clearly labelled. The upper panels show blots probed with an anti-Flag antibody to detect hPepT2 and its mutants, while the lower panels show blots probed with an anti-actin antibody as loading controls. The left panels show blots of total cell lysates, whereas the right panels show blots from the cell-surface biotinylation assay.
WT, wild type; NC, vector-transfected negative control. The numbers 214, 215, 342, 555, and 622 indicate the positions of the respective mutations in hPepT2.
