# Supplementary material for: Lateral opening site of human oligopeptide transporter 2 plays a key role in the interaction with polymyxins
Source: eLife. 2026 Oct 29;15:RP111387. doi: 10.7554/eLife.111387 (PMC13719170; doi:10.7554/eLife.111387)
Supplement: Peer review history

# Editor report: eLife Assessment

**DOI:** 10.7554/eLife.111387.3.sa0

**Authors:** Warren Andrew Andayi

**Affiliations:** Murang'a University of Technology Kenya

**Keywords:** Compelling, Fundamental

The study presents a **fundamental** structure interaction model describing how polymyxin interacts with hHepT2 the human oligopeptide transporter's 2 Aspartic acid residues to mediate nephrotoxicity via the D215 mutagenesis. These findings guided the rational design of potent polymyxin B analogues with reduced toxicity. The conclusions are supported by **compelling** evidence from computational and experimental data. The work will be of use to biochemists and medicinal chemists in developing the next generation of antibiotics for clinically relevant multidrug resistant bacteria.

# Referee report: Reviewer #1 (Public review)

**DOI:** 10.7554/eLife.111387.3.sa1

Summary:

Polymyxins are the last line of drugs to treat gram-negative bacteria induced multi-drug resistance, however, they cause nephrotoxicity in 60% of patients. In this work, authors have studied the structure-interaction relationship (SIR) of polymyxins with hPepT2 using computational and experimental methods. Moreover, it is observed that the electrostatic interactions coordinate the hPepT2-Polymyxin interactions, hence, an alanine scanning strategy is used to understand the interactions and derive the polymyxin variants.

Computational methods such as molecular modeling, coarse grained and all atom MD simulations, and interaction studies are performed. While the results are validated in the mouse model which is a great strategy to prove the hypothesis.

Strengths:

A clear understanding on the hPepT2-Polymyxin interactions and role of electrostatic interactions is one of the very important strengths of the paper. In addition, this work proposes a great pipeline for using computational approaches and experimental validation methods to guide the development of newer antibiotics.

Overall, the study proposes novel polymyxin analogues with reduced or no nephrotoxicity, thereby providing a promising foundation for the rational development of safer lipopeptide antibiotics.

Comment on revised manuscript:

I appreciate the effort by Authors to address my comments. The manuscript now contains details of ACE inhibitors in the introduction. In addition, method section is updated for better reproducibility of the MD simulation and structure modeling methods. I congratulate authors for reporting a wonderful scientific study. I don't have any further recommendations. Thank you!

# Referee report: Reviewer #3 (Public review)

**DOI:** 10.7554/eLife.111387.3.sa2

Summary:

Jiang et al. described findings aimed at interrogating the interactions of the antibiotic polymyxin B with human kidney proteins that mediate nephrotoxicity. Their findings using both computational molecular dynamics simulations and experimental approaches illustrate the importance of aspartic acid residues (D215) in mediating the antibiotic uptake into the cells, and upon mutagenesis with Alanine, the effects are less pronounced. Further, they could modify the antibiotic units interacting with proteins into less toxic peptides with retained antibacterial properties.

Strengths:

I was impressed by this text, which advances the knowledge of how the antibiotic causes human nephrotoxicity and how this could be exploited into less problematic antibiotic peptides.

Comments on revised version.

Majority of the raised issues were addressed satisfactorily.

# Author comment: Author response

**DOI:** 10.7554/eLife.111387.3.sa3

**Authors:** Xukai Jiang, Yining Luo, Mohammad AK Azad, Limei Xu, Min Xiao, Tony Velkov, Kade D Roberts, Visanu Thamlikitkul, Qi Tony Zhou, Fanfan Zhou, Jian Li

**Affiliations:** Shandong University Qingdao China; The University of Sydney Sydney Australia; Monash University Melbourne Australia; Shandong University Jinan China; Shandong University Jinan China; Monash University Clayton Australia; Monash University Melbourne Australia; Mahidol University Bangkok Thailand; Purdue University West Lafayette West Lafayette United States; The University of Sydney Sydney Australia; Monash University Clayton Australia

The following is the authors’ response to the original reviews.

**Reviewer #1 (Public review):**

(1) The introduction is well articulated; however, including a paragraph on the known inhibitors might be helpful in understanding the current status. In addition, it might also help to introduce Dabs, FADDI variants, Gly-sar and MIPS.

We thank the reviewer for the suggestion. We have included a paragraph on the substrates of hPepT2 (Lines 104-112).

(2) The following details of modeling with AlphaFold2 should be included: how the final structure was selected, what the RMSD and structure alignment of the template are, and the final selected structure. A section on modeling with all the parameter details might be useful for reproducing the structure. In addition, specify how the alanine scanning was performed alongside the structure prediction of polymyxins.

We appreciate the reviewer's careful assessment of our computational methodology. In the Materials and Methods section of the revised manuscript, we have added a dedicated subsection describing the detailed methods: "Structural modelling of hPepT2 and polymyxins" as shown below (Lines 435-447).

(1) hPepT2 structure prediction: The inward-open conformation of hPepT2 was predicted using AlphaFold2 via the ColabFold implementation with default parameters, employing the MMseqs2 multiple sequence alignment pipeline. To obtain the physiologically relevant outward-open conformation of hPepT2 for substrate binding, homology modeling was performed using MODELLER with the cryo-EM structure of rabbit PepT2 in the outward-open state (PDB: 7NQK) as the template. The sequence alignment between hPepT2 and rabbit PepT2 was conducted using Clustal Omega. One hundred models were generated, and the final model was selected based on the lowest Discrete Optimized Protein Energy score and verified by Ramachandran plot analysis (with >95% of residues in favored regions).

(2) Polymyxin structure and alanine scanning: The structure of polymyxin B1 was constructed and energy-minimized using the CHARMM36 force field. Computational alanine scanning of polymyxin B1 was performed by individually replacing each Dab (2,4-diaminobutyric acid) residue at positions 1, 3, 5, 8, and 9 with alanine using the mutagenesis wizard in PyMOL.

(3) In the all-atom MD simulation method, detailing several parameters might help in reproducing the results: simulation time for each system, water model, system composition, protonation state, box type and dimensions, salt ions and concentration, membrane parameters and ligand parameterization methods. Also, the following details on energy minimization might be useful: minimization algorithm, number of steps for minimization and structure restraints in place.

We thank the reviewer for the suggestion. In this study, all-atom molecular dynamics simulations were performed using the TIP3P water model for solvation. The system was placed in a rectangular simulation box with dimensions of 11 × 11 × 14 nm^3^. Neutralization was achieved by adding 0.15 M NaCl, resulting in a total of approximately 158,000 atoms. The membrane lipid composition consisted of 50% phosphatidylcholine, 25% phosphatidylethanolamine, and 25% phosphatidylserine, consistent with the coarse-grained molecular dynamics simulations. Force field parameters for polymyxin B1 were generated using CGenFF. Energy minimization was carried out using the steepest descent algorithm with default parameters. For production simulations, 500-ns simulations were run for sampling the outward‑open conformation, and 100-ns simulations were run for analyzing the polymyxin‑hPepT2 interaction. We have provided these methodological details in the revised manuscript (Lines 477-492).

(4) On page 6, line 210, the MIC is used for the first time; although MIC is given in the abbreviation list, the first occurrence should have a complete name. A one-line explanation of MIC in the introduction or wherever suitable might be better but is not mandatory.

We thank the reviewer for the suggestion. We have included the full term of MIC and its definition in the revised manuscript (Lines 236-238).

(5) Similarly, Gly-sar is first mentioned on page 8, line 301, but its complete name is only mentioned later on page 10, line 368. This can be addressed if a short description is included in the introduction section.

We apologize for the overlook. We have now referred to the full term of Gly-Sar and a short description on Page 3 (Lines 104-106), when it was first mentioned.

(6) For coarse-grained MD simulation, why were 2 replicates performed? Most studies perform 3 replicates, which are also good in terms of statistics and error bar calculations. In addition, the authors should specify whether an independent minimization is done for each of the two replicates or whether the minimization step is common for both.

In our study, coarse-grained MD simulations served as a preliminary exploration to identify the potential binding trajectory of polymyxin B to hPepT2, rather than the primary source of quantitative interaction data. The two independent CG-MD replicates yielded highly consistent final binding poses of polymyxin B at the lateral opening gate of hPepT2, serving this exploratory purpose. Furthermore, more detailed interaction energetics were subsequently characterized using all-atom MD simulations with four replicates (Fig. 4). We have now clarified in the revised Methods that independent energy minimization was performed for each of the two CG-MD replicates (Lines 465-469).

(7) For MD simulation results, giving simulation movies in supplementary results might be a better way to show how the trajectories behaved.

We thank the reviewer for the suggestion. We have prepared an animation of the coarse-grained MD trajectory showing the binding trajectory of polymyxin B to hPepT2, which has been submitted as a Supplementary Movie (.mp4 format). This movie visualizes how polymyxin B molecules spontaneously approach and bind to the lateral opening gate of hPepT2 over the 3-μs simulation. The Supplementary Movie is referenced in the revised Methods section (Lines 471-474).

(8) The description of visualisation software such as VMD or PyMol is missing. The authors should specify if any visualization tool is used.

We apologize for this omission. In the revised manuscript, we have specified in the Methods section that molecular visualizations, structural figures, and trajectory animations were prepared using PyMOL (Schrödinger) and ChimeraX (Lines 471-474).

(9) For the mouse model study, the authors claim that FADDI-795 has no observable nephrotoxicity; however, the n=3 shows that a very small number of mouse models were used to make the assumption. In addition, the number of mice used in each experiment is not explicitly mentioned in the methods section.

We have now added “n=3 each group” in the Methods section (Line 603). In this proof-of-concept study, we employed acute kidney damage analysis in a small number of mice to rapidly screen these analogues for nephrotoxicity. Three biological replicates were used because our mouse nephrotoxicity model is robust as demonstrated by a large number of animals in our drug discovery program (e.g. Nature Communications 2022, 13, 1625). Moreover, minimising animal use is a fundamental principle of the 3Rs (Replacement, Reduction, and Refinement).

(10) In Table 2, the column 8 header is not visible.

We apologize for the format error and have now fixed it in Table 2.

**Reviewer #1 (Recommendations for the authors):**

(1) The MD simulation trajectories or movies are not provided.

As mentioned in our response to Comment 7 above, a Supplementary Movie (mp4 format) showing the CG-MD binding pathway has been provided with the revised manuscript and referenced in the Methods section.

(2) Mouse model experiments are performed over n=3, which might not be statistically significant in case of an experiment with a large n.

Please see our response to Comment 9 above.

**Reviewer #2 (Public review):**

(1) Several conclusions would benefit from a more cautious interpretation. A major limitation is that several transporter mutations substantially altered total or membrane protein expression, making it difficult to distinguish effects on substrate binding from indirect effects caused by impaired transporter stability or trafficking. The authors acknowledge this limitation in the Discussion, but some mechanistic conclusions remain stronger than the available evidence supports.

We agree with the reviewer that it is difficult to draw definite conclusions when both K_m_ and V_max_ are altered in some mutants, especially when expression of the mutant transporter is impaired. However, we consider kinetic analysis to be more indicative of altered substrate binding, as changes in K_m_ are generally more closely associated with substrate recognition and binding, whereas reduced transporter expression predominantly affects V_max_. Nonetheless, to avoid overinterpretation, we have moderated our conclusions by replacing “was/is” with “may be” (such as Lines 318, 319, 323, and 347).

(2) Similarly, while the proposed binding model is biologically plausible and supported by mutagenesis, it remains an inferred model derived from molecular simulations rather than a direct structural determination. Statements describing the model as "validated" should therefore be moderated to indicate that the experimental data provide support rather than definitive structural confirmation.

We thank the reviewer for the suggestion and have replaced “validated” with “explored” in the main text (Lines 298).

(3) The translational implications are promising but remain preliminary. Although FADDI-795 demonstrated reduced nephrotoxicity in the mouse model while maintaining antibacterial activity, no pharmacokinetic studies were presented to demonstrate reduced renal accumulation or altered tissue distribution, and additional efficacy studies in infection models would further strengthen the therapeutic claims.

We thank the reviewer for the suggestion. The current study is a proof-of-concept investigation to determine whether the structure-interaction relationship (SIR) model of hPepT2 and polymyxin can be used to design new lipopeptides with reduced nephrotoxicity. Following screening of the newly designed lipopeptides, we will select the most promising candidate for comprehensive pharmacological evaluations, including pharmacokinetics, toxicology, and efficacy.

**Reviewer #2 (Recommendations for the authors):**

(1) Strengthen the interpretation of the mutagenesis data. For transporter mutants that exhibit reduced total or cell surface expression, consider discussing more explicitly the extent to which reduced polymyxin uptake may result from altered protein stability or trafficking rather than direct effects on substrate recognition. Where possible, clarify this distinction throughout the Results and Discussion.

We thank the reviewer for the recommendation. As suggested, we have expanded the discussion on the mechanisms underlying the altered protein expression of the hPepT2 mutants (Lines 325-333).

(2) Moderate statements regarding structural validation. The molecular dynamics simulations, mutagenesis, and functional studies provide strong support for the proposed structure-interaction model; however, wording such as "validated the first SIR model" could be softened to reflect that the binding model remains computationally inferred rather than directly resolved by structural methods.

We thank the reviewer for the recommendation. We have revised the statement as described in our response to Comment 2 above.

(3) Expand the discussion of study limitations. A more explicit discussion of the limitations associated with molecular dynamics predictions, the influence of altered transporter expression on functional interpretation, and the lack of direct pharmacokinetic measurements of renal accumulation would improve the balance of the manuscript.

We thank the reviewer for the recommendation. We have expanded the discussion by elaborating the limitations of the molecular dynamics predictions (Lines 289-291), the effect of altered transporter expression on functional interpretation (Lines 324-333), and the lack of direct pharmacokinetic and renal accumulation measurements (Lines 393-401).

(4) Provide additional methodological detail where appropriate. Please clarify the number of biological replicates used for each experimental approach, report exact P values where practical, and indicate whether assumptions for the statistical tests were assessed.

We thank the reviewer for the recommendation. We have provided more information on experimental replicates in the figure legends. *P* values have been added (Lines 208-217), and the statistical tests used for every analysis are now specified in the corresponding table or figure legends.

(5) Future validation of the lead compound. Although this may fall outside the scope of the present manuscript, it would be helpful to briefly discuss future studies aimed at evaluating the pharmacokinetics, renal exposure, and efficacy of FADDI-795 in relevant infection models, as these will be important for establishing its translational potential.

We thank the reviewer for the recommendation. We have now incorporated this discussion into the revised manuscript (Lines 396-399).

(6) Improve figure presentation. Several figure legends could include additional methodological information to make the figures more self-contained. In particular, indicating sample sizes, statistical tests used, and definitions of error bars would improve readability.

We thank the reviewer for the recommendation. We have added this information to the figure legends as suggested.

(7) Language and style. The manuscript would benefit from careful language editing to improve grammar, sentence structure, and readability. Several long sentences in the Discussion could be shortened, and minor typographical errors should be corrected throughout the manuscript.

We thank the reviewer for the recommendation. We have revised the long sentences where possible and carefully proofread the manuscript to improve grammar, sentence structure, and readability. The revised manuscript has also been reviewed by a native English speaker.

(8) Terminology. Please ensure that abbreviations such as "SIR" are defined at first use and are used consistently throughout the manuscript.

We have confirmed that SIR is defined when first introduced in both the Abstract and main text (Lines 47 and 120).

(9) Minor corrections.

- Check for consistency in the naming of hPepT2/hPEPT2 throughout the manuscript.

- Verify that all figures, supplementary figures, and tables are cited sequentially in the text.

- Consider reporting confidence intervals alongside kinetic parameters (K_m_ and V_max_) where appropriate to facilitate interpretation.

We thank the reviewer for the recommendation. We have revised the manuscript accordingly. “hPepT2” is now used consistently throughout the text. We have revised the order of the supplementary figures to ensure that they are cited sequentially in the text; and included the 95% confidence interval of the K_m_ and V_max_ values of the D215A mutant compared to the wild type (Lines 208-212).

**Reviewer #3 (Public review):**

(1) Interactions of Polymyxin B with kidney proteins were not demonstrable in vivo, and with reliable technologies such as X-ray or NMR.

We thank the reviewer for the comment. To the best of our knowledge, polymyxin-induced nephrotoxicity is primarily driven by renal tubular accumulation, followed by mitochondrial dysfunction, oxidative stress, inflammatory activation, and apoptosis, ultimately resulting in proximal tubular injury. Little is known about the molecular interactions of polymyxins with kidney proteins.

**Reviewer #3 (Recommendations for the authors):**

(1) Title: I suggest that you bring out the actual findings to convey the message.

We thank the reviewer for the comment. We have changed the title to “Lateral opening site of human oligopeptide transporter 2 plays a key role in the interaction with polymyxins”.

(2) Line 133: Provide data for these 10 molecules (possibly by supplementary file/table).

We thank the reviewer for the suggestion. As described in the Methods, ten coarse-grained polymyxin molecules were randomly inserted into the upper water layer of the simulation system (Lines 452-456). Because these molecules are structurally identical and share the same force field parameters, they differ only in their initial random positions. Thus, we believe that presenting the coordinates or labelling the individual molecules would not provide additional information and can be misleading.

(3) Line 135: Figure S3 mentioned prior Figure S2.

We apologize for the error. We have now revised the sequence of the supplementary figures.

(4) Lines 159 - 171: Cite Figure 5 a - b appropriately. Describe the obtained results fully.

We thank the reviewer for the suggestion. We have expanded the relevant section to include a more detailed description of the results, with reference to Fig. 5a and 5b (Lines 187-193).

(5) Line 208: Was cytotoxicity performed to validate this claim?

We did not specifically evaluate the cytotoxicity of the lipopeptides in cultured cells because the cellular uptake data already indicated their potential toxicity. Importantly, histopathological assessment in animal models provides the most reliable evaluation of their in vivo toxicity (Nature Communications 2022, 13, 1625).

(6) Line 509: Provide the approval number.

The animal ethics approval number is AEC37419, which has been added to the “Ethics approval and consent to participate” section.

(7) Table 2: Add the Standard deviation and the number of replicates performed.

We thank the reviewer for the suggestion. We have added “n = 3 mice per group” to the table legend. As described in the Methods (Lines 590-597), the MIC of each lipopeptide was determined. MIC values are reported as absolute concentrations rather than continuous variables; therefore, reporting the mean ± standard deviation is not applicable. Likewise, the maximum kidney Semi-Quantitative Score (SQS) was determined by histological examination and is presented as the representative maximum score for each treatment group. Accordingly, the calculation of a mean and standard deviation is not appropriate for either MIC or SQS values presented in Table 2.

(8) The authors need to indicate the number of replicates performed in the methods section.

We thank the reviewer for the suggestion. As mentioned in our response to Recommendation 6 from Reviewer 2 above, we have added this information to the figure legends as suggested.
